# De novo designed protein inhibitors of amyloid aggregation and seeding

**DOI:** 10.1073/pnas.2206240119

**Published:** 2022-08-15

**Authors:** Kevin A. Murray, Carolyn J. Hu, Sarah L. Griner, Hope Pan, Jeannette T. Bowler, Romany Abskharon, Gregory M. Rosenberg, Xinyi Cheng, Paul M. Seidler, David S. Eisenberg

**Affiliations:** ^a^Department of Chemistry and Biochemistry, University of California, Los Angeles, Los Angeles, CA 90095-1569;; ^b^Department of Biological Chemistry, University of California, Los Angeles–Department of Energy Institute, University of California, Los Angeles, Los Angeles, CA 90095-1737;; ^c^Molecular Biology Institute, University of California, Los Angeles, Los Angeles, CA 90095-1570;; ^d^HHMI, University of California, Los Angeles, Los Angeles, CA 90095-1570;; ^e^Department of Pharmacology and Pharmaceutical Sciences, University of Southern California, Los Angeles, CA 90089-9121

**Keywords:** protein design, amyloid, tau, alpha-synuclein, amyloid-beta

## Abstract

Neurodegenerative diseases are characterized by the pathologic accumulation of aggregated proteins. Known as amyloid, these fibrillar aggregates include proteins such as tau and amyloid-β (Aβ) in Alzheimer’s disease (AD) and alpha-synuclein (αSyn) in Parkinson’s disease (PD). The development and spread of amyloid fibrils within the brain correlates with disease onset and progression, and inhibiting amyloid formation is a possible route toward therapeutic development. Recent advances have enabled the determination of amyloid fibril structures to atomic-level resolution, improving the possibility of structure-based inhibitor design. In this work, we use these amyloid structures to design inhibitors that bind to the ends of fibrils, “capping” them so as to prevent further growth. Using de novo protein design, we develop a library of miniprotein inhibitors of 35 to 48 residues that target the amyloid structures of tau, Aβ, and αSyn. Biophysical characterization of top in silico designed inhibitors shows they form stable folds, have no sequence similarity to naturally occurring proteins, and specifically prevent the aggregation of their targeted amyloid-prone proteins in vitro. The inhibitors also prevent the seeded aggregation and toxicity of fibrils in cells. In vivo evaluation reveals their ability to reduce aggregation and rescue motor deficits in *Caenorhabditis elegans* models of PD and AD.

## Significance

We have investigated the usefulness of de novo designed miniproteins, 35 to 48 amino acid residues in length, for the inhibition of protein fibrils associated with numerous neurodegenerative diseases. From known atomic structures of fibrils of tau, alpha-synuclein, and amyloid-beta, we designed miniproteins to cap the growing tips of fibrils, halting further growth. We find the miniproteins halt protein aggregation into fibrils and halt the ability of fibrils to induce or “seed” fibril growth in other cells. A miniprotein that inhibits aggregation of its target protein is specific and does not inhibit aggregation of other proteins. An advantage of miniproteins as eventual therapeutics is that they can be genetically encoded and possibly delivered to diseased brains by viral vectors.

The aberrant aggregation of proteins into amyloid fibrils is a hallmark of many neurodegenerative diseases, including Alzheimer’s disease (AD) and Parkinson’s disease (PD) (1). In AD, amyloid-β (Aβ) and tau amyloid fibrils comprise the extracellular amyloid plaques and intracellular neurofibrillary tangles, respectively, characteristic of disease progression (2). Likewise, intracellular Lewy bodies found in the neurons of patients with PD and dementia with Lewy bodies (DLB) are primarily composed of αSyn fibrils (3). There are currently no therapies capable of significantly slowing or stopping the progression of any of these diseases, and inhibition of fibril formation has become a major target for therapeutic development (4, 5). Amyloid fibrils are composed of repeating layers of β-strand–rich protein monomers stacked upon each other, forming β-sheets. The β-sheets interdigitate to form a stable fibril core through interactions known as steric zippers (6). Antiamyloid therapies have typically focused on small molecules that prevent aggregation or dissociate preexisting aggregates and antibodies that promote fibril clearance (7–9). An alternative approach is the design of molecules that bind to the ends of the growing fibrils, capping their growth and preventing the further addition of more protein monomers. This approach has been successfully used to design peptide-based inhibitors of tau, Aβ, and αSyn aggregation (10–14). This design strategy considers the atomic structures of fibrils, employing rational and computational design techniques to derive a peptide sequence complementary to the growing fibril surface.

Since the initial designs of structure-based capping inhibitor peptides, many advances have been made in both the determination of amyloid protein structure, as well as in methods of protein design. The first atomic-resolution structures of amyloid fibrils determined by X-ray crystallography were restricted to small peptide segments ∼6 to 11 amino acids in length (15). The recent advent of cryoelectron microscopy (cryo-EM), microelectron diffraction (MicroED), and solid-state NMR (ssNMR) spectroscopy have enabled the determination of amyloid protein structures that were previously unsolvable (16–18). These techniques have been used to solve an ever-growing list of structures of both recombinantly derived fibrils (19–21) as well as fibrils directly extracted from patient tissue (22–29). These structures have provided key insights into fibril architecture and polymorphism in relation to disease.

Like the structural knowledge of amyloid fibrils, the toolbox of protein structure prediction and design has been rapidly expanding in recent years (30, 31). Significant advances in algorithms and computing power have facilitated the de novo design of proteins with a variety of properties and functions, ranging from stability, pH sensitivity, to even logic operations, with vast potential for use in therapeutics, diagnostics, etc (32–36). While the underlying design principles of de novo generated proteins are becoming well established, examples of their direct application into biological systems are still limited. In this work, we use de novo protein design to create 35 to 50 residue miniproteins that bind to the growing ends of tau, αSyn, and Aβ fibrils. We target recently determined full-length atomic structures of each amyloid protein in our designs to generate miniproteins capable of inhibiting aggregation, seeding, and toxicity both in vitro and in vivo.

## Results

### High-Throughput Computational Design Pipeline.

**Fig. 1.**
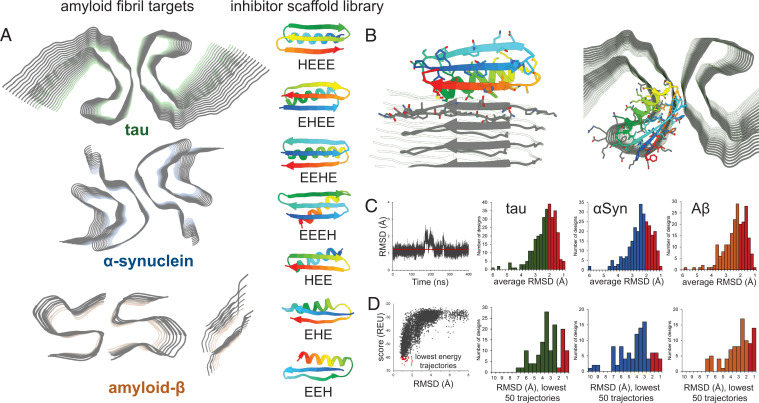
Computational design of amyloid-inhibiting miniproteins. (*A*) Amyloid fibrils of three different proteins were used as scaffolds for inhibitor design: tau, αSyn, and Aβ. Atomic structures of the tau paired helical filament derived from Alzheimer’s disease patient brain (*Top*) (22), the αSyn rod polymorph (*Middle*) (20), and two forms of Aβ (*Bottom*) were targeted (14, 41). A library of de novo designed miniproteins was used as inhibitor scaffolds. Seven unique classes of inhibitors were used, each class differing from the arrangement of secondary structural elements (H = α-helix; E = β-sheet). (*B*) Inhibitor scaffolds were docked to the ends of the fibril structures, capping their growth by preventing further addition of protein monomers. Binding of the miniprotein scaffolds to the fibrils was primarily driven by interacting β-sheets, mimicking the fibril native stacking. (*C*) The stabilities of top-ranking hits from the docking calculations were assessed by long-range molecular dynamics simulations. Those inhibitors with the lowest average rmsds over time were selected for further testing (red bars). (*D*) Final screening of inhibitors was performed using Rosetta’s ab initio structure prediction algorithm. The structures of each inhibitor were predicted based on primary sequence alone. The energies of each prediction trajectory were plotted against their rmsd to the original design. Those inhibitors whose lowest energy predictions were the smallest rmsd from the original design were then selected for experimental characterization (red bars).

Three amyloid proteins were chosen for inhibitor design: tau, αSyn, and Aβ (Fig. 1 *A*, *Left*). Atomic-level fibril structures of all these proteins have been determined, including short peptide segments determined through crystallographic methods and full-length structures solved by cryo-EM/ssNMR. For tau, the paired helical filament (PHF) structure derived from AD patient brains was selected (22). The PHF structure is the dominant tau fibril morphology in AD brains and this structure is conserved in fibrils extracted from the brains of different patients (37). For αSyn, the rod polymorph from Li et al. was selected (20). Cryo-EM structures of αSyn reported from multiple laboratories have demonstrated the rod polymorph to be the dominant fibril morphology (38–40). A recent study of brain-derived αSyn fibrils shows that the core structural elements of the αSyn rod polymorph are still maintained in patients with the synucleinopathy multiple system atrophy (MSA) (25). For Aβ, two structures were used for inhibitor design. One is the disease-relevant full-length structure from Riek and coworkers (41), the other is a segment from amyloid β (residues 16 to 23) with the D23N Iowa mutation, which has been successfully used to design peptide-based inhibitors (14).

The design of the inhibitor library was performed using the software suite Rosetta. Backbone topologies were generated using the blueprint format of RosettaRemodel to guide Rosetta’s Monte Carlo–based fragment assembly, followed by the FastDesign algorithm to generate the amino acid sequence (42, 43). For the inhibitor scaffolds, miniproteins, 35 to 50 amino acids in length, were designed into seven unique classes, with each class differing by the arrangement of secondary structural elements (Fig. 1 *A*, *Right*). Each inhibitor topology contains one α-helix (H) and two or three β-strands (E), yielding the seven classes: HEE, EHE, EEH, HEEE, EHEE, EEHE, and EEEH. To mimic the natural interactions found in amyloid fibrils, the primary interaction between the inhibitors and fibrils is the stacking of a β-strand of the inhibitor onto the β-strand of the growing fibril end (Fig. 1*B*). Because of this, only inhibitor topologies with at least one β-strand were selected. Several classes of inhibitors composed exclusively of β-sheets (EEE-EEE) were tested, but did not yield consistently stable designs, indicating the importance of the stabilizing α-helix found in each of the selected classes. In addition, as the final aim of these inhibitors is to therapeutically target largely intracellular protein aggregates, disulfide bonds were not incorporated into the designs, as has been done in previous studies, because of the reducing conditions of the cytoplasm (44). A total of 5,000 unique scaffolds were generated for each inhibitor class. Inhibitors were docked onto the fibrils using Rosetta’s MotifGraft protocol, creating a backbone alignment of a selected portion of the native fibril strand with the β-strand of the inhibitor (45). Once docked to the fibril, the inhibitor sequence along the binding interface was optimized to increase binding energy.

**Fig. 2.**
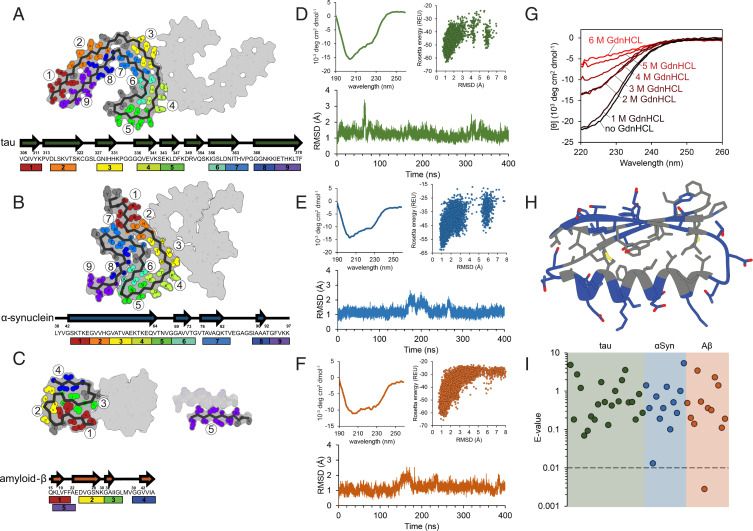
Biophysical characterization of designed inhibitors. (*A*–*C*) Multiple binding sites on each amyloid fibril structure were selected for targeted inhibitor design. The inhibitor scaffolds were systematically docked to different sites along the fibril ends (each number/color corresponding to a unique binding site). The chosen binding sites correspond to particular β-strand segments (shown in arrows) occurring along the protein chain for tau (*A*) αSyn (*B*), and Aβ (*C*). (*D*–*F*) Initial biophysical characterization of each design consisted of CD spectroscopy (*Top Left*), ab initio structure prediction (*Top Right*), and long-range molecular dynamics simulations (*Bottom*). Inhibitors iTau-N (*D*), i αSyn-F (*E*), and iAβ-H (*F*) are shown to maintain stable folds both computationally and experimentally. (*G*) To assess the stability of the designed inhibitors, CD measurements were taken after a 20-min incubation with increasing concentrations of the denaturant GdnHCl. iTau-N (shown) remains completely stable in 1 M GdnHCl. GdnHCl denaturation curves do not necessarily show a full cooperative unfolding transition, but may indicate destabilization of the folded miniprotein. (*H*) The fold of each designed inhibitor is driven by a hydrophobic core region (gray residues) surrounded by an exterior of charged and polar residues (blue) (inhibitor iTau-N shown). (*I*) Each inhibitor was generated de novo, with no apparent homology to known naturally occurring proteins. BLAST E-values, a metric indicating protein homology, demonstrating the designs are well above the significance threshold of 0.01, for all inhibitors targeting tau (green), αSyn (blue), and Aβ (orange), with the exception of iAβ-L.

The tips of each amyloid fibril structure used in this study are nearly flat, open β-strand–rich surfaces. Because of this, no obvious binding cavity exists, and it is unclear whether any particular segment of the surface is the most important for fibril aggregation. Because of this, we chose to systemically select each possible segment of the fibril ends as an inhibitor binding site (Fig. 2 *A*–*C*). Nine, nine, and five unique sites were selected for the tau, αSyn, and Aβ structures, respectively. Inhibitors from each of the seven classes were docked and sequence optimized to each site in an all-vs.-all fashion, yielding ∼1 million unique inhibitor sequences. Docked poses were ranked by several scoring metrics pertaining to the stability of the inhibitor/fibril interaction, including ddg (binding energy), the number of unsaturated hydrogen bonds, and number of atoms in the interface. Additionally, metrics assessing the stability of the inhibitor alone were used, such as total score and p_aa_p (empiric probability of amino acid at a certain position based on backbone dihedral angles). These metrics were demonstrated to be predictive of successful designs in work by Chevalier et al. to create miniprotein binders (44).

To further validate the fold and stability of each inhibitor, several more rigorous computational steps were incorporated into the screening pipeline. First, long-range molecular dynamics (MD) simulations of unbound inhibitors were performed to measure stability in a dynamic system (Fig. 1*C*). Simulations in an explicit cubic water box were carried out in GROMACS 2018 for 200 ns (46). Those inhibitors that showed stability after the initial MD run, as measured by a low rmsd from the starting configuration, were tested for an additional 400 ns. The folds of the most stable inhibitor designs were then subjected to Rosetta’s fragment-based ab initio folding algorithm, which predicts protein structure based on primary sequence (47). The sequences of top-ranking inhibitor designs were provided to the prediction algorithm, and 50,000 trajectories were calculated per sequence. The lowest energy trajectories converging on a conformation close in backbone rmsd to the original design indicates a stable fold (Fig. 1*D*). Inhibitors whose 50 lowest energy trajectories had the lowest rmsd values were then selected for experimental characterization.

### Biophysical Characterization of Inhibitor Proteins.

From the top-ranking inhibitor sequences selected for experimental testing, 46 soluble miniproteins were expressed and purified (*SI Appendix*, Tables 1 and 2). The inhibitors were expressed in *Escherichia coli* with a thrombin-cleavable N-terminal His-tag and purified using a Ni-NTA affinity column followed by size-exclusion chromatography (*SI Appendix*, *Materials and Methods*). Beyond computational prediction, circular dichroism (CD) spectroscopy was used to verify that each miniprotein adopts a stable fold (Fig. 2 *D*–*F* and *SI Appendix*, Fig. 1). As seen in *SI Appendix*, Fig. 1*D*, analysis of CD spectra of select purified miniproteins shows a mixed distribution of alpha-helical, antiparallel beta-sheets, and loops, all of which are consistent with their computational designs (59). One selected tau inhibitor, iTau-N, was treated with increasing concentrations of the denaturant guanidinium hydrochloride (GdnHCl) to assess its stability (Fig. 2*G*). No significant changes in its CD spectrum were observed at 1 M GdnHCl, and the CD signal gradually diminishes as GdnHCl concentration increases past 1 M. Lacking any disulfide bonds, the stability of each inhibitor fold is likely derived from its tightly packed hydrophobic core, with charged and polar residues decorating the miniprotein exterior (Fig. 2*H*). The sequences of each tested inhibitor are truly de novo, lacking apparent homology to natural protein sequences. BLAST E-values, the statistical term indicating sequence homology, for each inhibitor are well below the significance threshold, except for iAβ-F, which holds some happenstantial sequence similarity to a universal stress family protein from *Pediococcus claussenii* (Fig. 2*I*) (48).

**Fig. 3.**
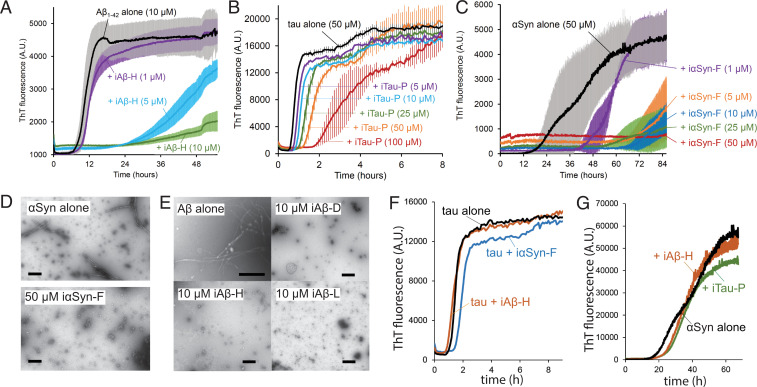
Inhibitors prevent amyloid aggregation by capping fibril ends. (*A*–*C*) Thioflavin T aggregation kinetics assays for Aβ (*A*), tau (*B*), and αSyn (*C*). Each amyloid protein was aggregated alone and in the presence of increasing concentrations of inhibitor, resulting in reduction in the rate of aggregation or complete abolition of aggregation. (*A*) The aggregation of Aβ_1–42_ (10 µM monomer) with the inhibitor iAβ-H. (*B*) Aggregation of αSyn (50 µM monomer) with inhibitor iαSyn-F nearly eliminates measured aggregation, even at substoichiometric ratios. (*C*) Tau k18+ (50 µM monomer) with the inhibitor iTau-P (100 µM) produces a fourfold increase in aggregation lag time. (*D*) Transmission electron micrographs (TEMs) of αSyn (50 µM) aggregated in the absence (*Top*) or presence (*Bottom*) of iαSyn-F show the inhibitor prevents the formation of fibrillar aggregates. (Scale bar, 50 nm.) (*E*) TEM images of Aβ_1–42_ alone (10 µM) reveal numerous fibrils, whose growth is prevented by the addition of equimolar amounts of iAβ-D, iAβ-H, and iAβ-L. (Scale bar, 100 nm.) (*F*) Both iαSyn-F and iAβ-H show little effect on tau k18+ aggregation (50 µM k18+ monomer, 50 µM inhibitors). Likewise, iTau-N and iAβ-H have minimal influence on αSyn aggregation (50 µM αSyn monomer, 50 µM inhibitors). All ThT experiments were performed with *n* = 3 experimental replicates.

**Fig. 4.**
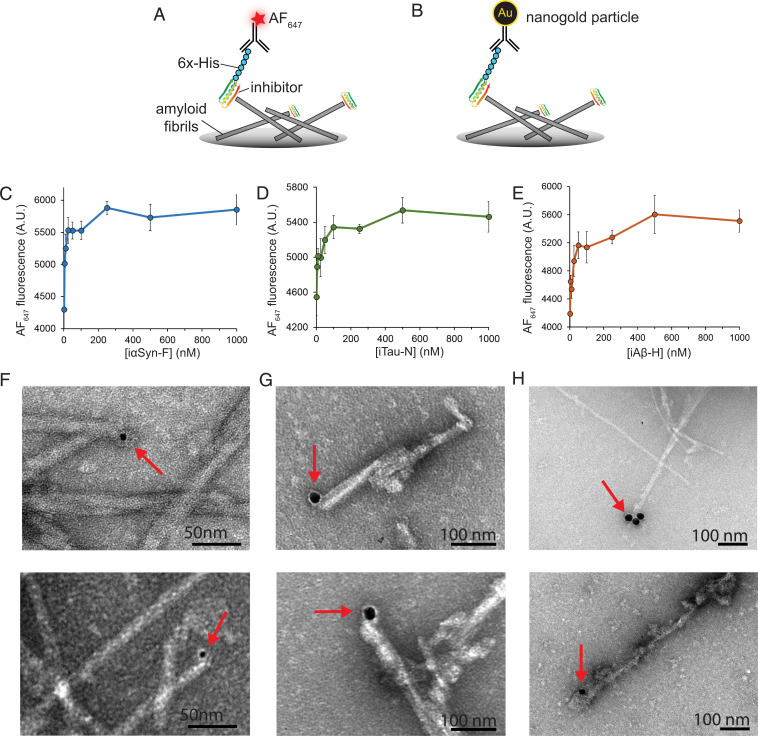
Binding of designed inhibitors to target amyloid fibrils. (*A*) ELISA binding assays were used to measure inhibitor binding to fibril ends. Inhibitors with uncleaved His-tags were incubated with fibril-coated plates, then labeled with an AF_647_-conjugated anti-His primary antibody. Fluorescence measurements at different concentrations of inhibitor (iTau-N shown) illustrate binding saturation in the high nanomolar regime. (*B*) To visualize the designed miniproteins bound to the fibril ends, inhibitors were incubated with fibrils, then labeled with 5 or 20 nm gold nanoparticles (indicated by red arrows), demonstrating a binding mode consistent with their intended design. (*C*–*E*) ELISA binding curves of iαSyn-F to αSyn fibrils (*C*), iTau-N to tau k18+ fibrils seeded with AD brain-derived fibrils (*D*), and iAβ-H to Aβ_1–42_ fibrils (*E*). (*F*–*H*) Transmission electron micrographs of amyloid fibrils bound with miniprotein inhibitors labeled with gold nanoparticles. (*F*) iαSyn-E bound to αSyn fibrils. (*G*) iTau-N bound to tau k18+ fibrils seeded with AD brain-derived fibrils. (*H*) iAβ-H bound to Aβ_1–42_ fibrils. In all three samples, inhibitors are observed binding primarily to the tips of the fibrils, consistent with their intended design. Additional TEM images are available in *SI Appendix*, Fig. 5.

We next sought to assess the effects of the inhibitors on primary amyloid aggregation. Thioflavin T (ThT) kinetics assays were performed for tau, αSyn, and Aβ in the presence of inhibitors. For the tau assays, monomeric tau k18+ (Q244–E380), which contains the core observed in the PHF structure (22), was used at 50-µM concentration. For αSyn, 50 µM of full-length αSyn was used, and for Aβ, 10 µM of Aβ_1–42_ was used. Screening of each inhibitor with its designed target (*SI Appendix*, Figs. 2–4) identified constructs capable of completely abolishing or significantly delaying aggregation. At equimolar ratios, the Aβ inhibitor iAβ-H prevents the aggregation of Aβ_1–42_ (Fig. 3*A*). At a 2:1 stoichiometric ratio (inhibitor/tau monomer), the inhibitor iTau-P leads to a fourfold increase in the lag time needed for tau k18+ to aggregate (Fig. 3*B*). The αSyn inhibitor iαSyn-F prevents αSyn aggregation at even substoichiometric ratios, with only minimal aggregation observed at 1:5 inhibitor to αSyn ratio (Fig. 3*C*). Negative stain transmission electron micrographs of αSyn and Aβ_1–42_ illustrate that iαSyn-F and iAβ-H fully prevent fibril formation in vitro (Fig. 3 *D* and *E*). The effects of each inhibitor are also specific to the amyloid protein they are designed to target. iαSyn-F and iAβ-H have little effect on tau k18+ aggregation, while iTau-P and iAβ-H show little effect on αSyn aggregation (Fig. 3 *F* and *G*).

**Fig. 5.**
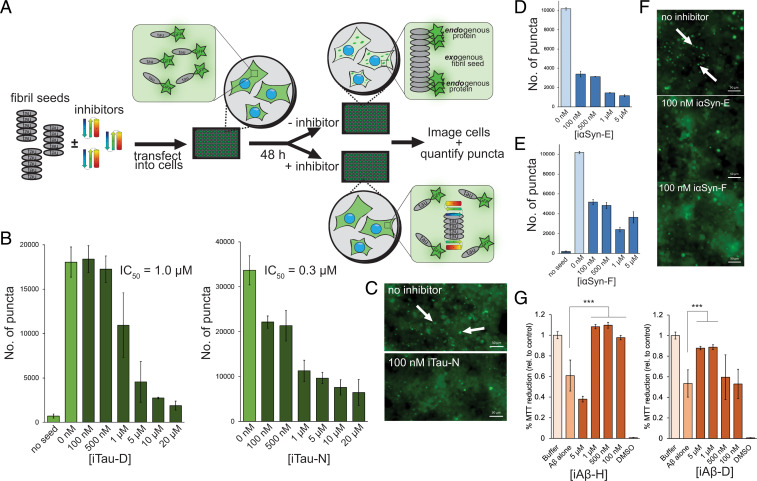
Inhibition of amyloid seeding and toxicity in cells. (*A*) To assess the effects of the designed inhibitors on amyloid seeding within cells, a HEK293T biosensor cell assay was used for both tau and αSyn. Fibril seeds (tau shown) are incubated overnight with inhibitors, then transfected into the biosensor cells. The cells overexpress amyloid protein (either tau k18 or αSyn) fused to YFP/CFP. At baseline, the cells show diffuse fluorescence, but the endogenous fluorescent amyloid protein can be incorporated into the transfected exogenous fibril seeds, resulting in visible fluorescent puncta. Addition of inhibitor caps the fibrils, preventing the incorporation of the YFP/CFP-labeled amyloid protein and the subsequent formation of puncta. (*B*–*E*). Inhibitors of tau and alpha-synuclein inhibit intracellular seeding in biosensor cells. Number of puncta on the *y* axis refers to number of fluorescent intracellular aggregates per experimental well of a 96-well plate. (*B*). Inhibitors iTau-D and iTau-N cause a significant reduction in the number of fluorescent puncta in cells transfected with AD patient brain extract containing tau fibrils. Minimal aggregation occurs in the absence of transfected fibril seeds. (*C*) Fluorescent microscopy images of biosensor cells with and without 100 nM iTau-N. Seeded aggregates can be visualized by discrete bright puncta (indicated by white arrows). (*D*–*F*) Inhibitors iαSyn-E (*D*) and iαSyn-F (*E*) greatly reduce aggregated puncta in biosensor cells expressing fluorescently labeled αSyn. (*F*) Similar to tau aggregates, αSyn aggregates can be visualized as intracellular fluorescent puncta and quantified (white arrows). (*G*) MTT dye reduction assays were used to assess the capacity of inhibitors to mitigate Aβ aggregate-induced cytotoxicity in N2a neuronal cells. Aβ aggregates alone (1 µM) resulted in ∼40% cell death compared to buffer control. This toxicity can be rescued by addition of either iAβ-H or iAβ-D. (Scale bars, 50 µm.) All error bars represent SD. All statistical analyses were performed using a one-way Analysis of Variance (ANOVA) (***P = 0.002; N.S., nonsignificant).

To gain an approximate measurement of binding affinity each inhibitor has for its target fibrils, we performed enzyme-linked immunosobent assay (ELISA) binding assays. Fibrils of tau k18+ seeded with AD brain fibrils, αSyn, or Aβ were coated onto the bottom of a plate, then incubated with inhibitors with their N-terminal His-tag uncleaved. Following this, an anti-His antibody conjugated with an Alexa Fluor 647 fluorescent dye was added (Fig. 4*A*). Fluorescent measurements of the samples show the inhibitors reach a binding saturation in the low-hundreds nanomolar range (Fig. 4 *C*–*E*). To validate that the binding to the inhibitors truly occurs at the fibril tips, we performed a nanogold binding assay in conjunction with electron microscopy. Similar to the ELISA, an EM grid was coated with amyloid fibril, then treated with inhibitors with their His-tag intact. The sample was then treated with an anti-His primary antibody followed by secondary antibody conjugated to a gold nanoparticle, which is highly visible by EM (Fig. 4*B*). Recombinant tau k18+ fibrils seeded with AD brain-derived tau fibrils were incubated with inhibitor iTau-N (Fig. 4*F*), αSyn fibrils incubated with iαSyn-E, and Aβ_1–42_ fibrils incubated with iAβ-H. A total of 15 to 20 EM images were taken for each experimental condition, with representative images displayed in Fig. 4 *F*–*H*. Gold nanoparticles complexed to inhibitors can be seen binding to the tips of their target fibrils (additional images in *SI Appendix*, Fig. 5), highlighting that binding to the fibrils is consistent with the intended design.

### Effects of Inhibitors on Amyloid Seeding and Toxicity.

**Fig. 6.**
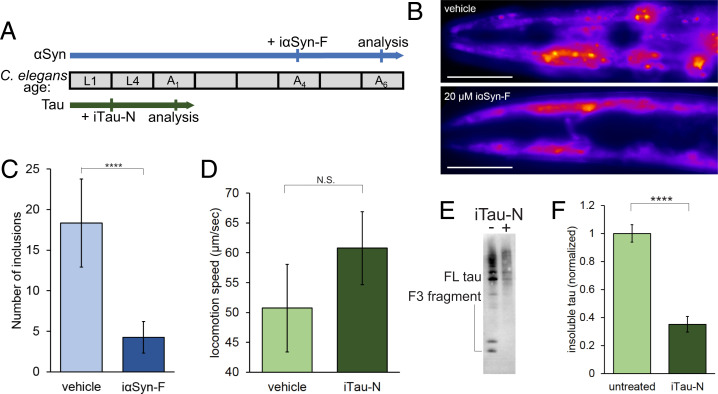
Inhibitors prevent amyloid aggregation in *C. elegans* model strains. (*A*) Two strains of *C. elegans* were used to test the in vivo efficacy of the designed miniprotein inhibitors (αSyn: DDP1, tau: BR5706). A timeline of worm lifespan through larval stages (L) and adult days (A) is shown, indicating age of worms during inhibitor treatment and subsequent analysis. (*B* and *C*). Alpha-synuclein strain DDP1 overexpresses fluorescently labeled αSyn, which aggregates in the worm head region as adults. (*B*) Vehicle-treated worms display numerous fluorescent puncta as day 6 adults, which are diminished with the addition of iαSyn-F. (Scale bars, 100 µm.) (*C*) Quantification of head region αSyn aggregates reveals a large reduction in inhibitor-treated worms. *n* = 15 worms were used for each experimental condition. (*D*–*F*). Tau strain BR5706 coexpresses full-length tau with the proaggregation V337M mutation and the F3 fragment of the tau microtubule binding domain with the proaggregation K280 deletion. (*D*). The movement of treated tau worms was tracked, and average locomotion speed across 30-s intervals was measured. Treatment iTau-N leads to an increase in speed, indicating a recovery of locomotion deficits. *n* = 15 worms were used for each experimental condition. (*E*) Tau Western blot of RIPA-soluble worm extracts with and without inhibitor iTau-N treatment. Bands of full-length (FL) and the F3 fragment are indicated. (*F*) Western blot quantification shows a significant reduction of insoluble tau species in the iTau-N–treated *C. elegans* compared to vehicle control. Experiment was performed in triplicate, with *n* > 100 worms for each experimental condition. All error bars represent SD. All statistical analyses were performed using an unpaired *t* test (****P < 0.0001; N.S., nonsignificant).

Amyloid pathology is believed to spread throughout the brain via a process known as templated seeding. Fibrillar aggregates that form in one cell migrate to adjacent cells and seed the subsequent aggregation of additional soluble protein. Having demonstrated the effects of the designed inhibitors on primary amyloid aggregation in vitro, we next aimed to assess their ability to prevent seeding in cells. HEK293T biosensor cells overexpressing either tau k18 or αSyn fused with either yellow or cyan fluorescent protein (YFP, CFP) were used in the seeding assays (Fig. 5*A*). In nontreated cells, the endogenous YFP/CFP-fused amyloid proteins remain soluble and unaggregated, visible as diffuse fluorescence throughout the cell. Upon the addition of an exogenous fibril seed, either recombinant or brain derived, the fluorescent endogenous protein becomes incorporated into the fibrillar form. This is visualized as bright fluorescent puncta forming within the cell. For tau seeding, AD patient brain extract was incubated with each inhibitor (10 µM final inhibitor concentration) overnight; then the mixture was applied to the tau biosensor cells. Forty-eight hours later, the cells were imaged and the number of fluorescent puncta were quantified (*SI Appendix*, Fig. 6). Several inhibitors showed a significant reduction in the number of seeded puncta, including iTau-D and iTau-N (Fig. 5 *B* and *C*), with inhibitory effects observed in the nanomolar range. Similarly, seeding αSyn biosensor cells with recombinant αSyn fibrils incubated with the panel of αSyn inhibitors identified many inhibitors with inhibitory effects (*SI Appendix*, Fig. 7), including iαSyn-E and iαSyn-F (Fig. 5 *D*–*F*), both of which also show nanomolar range efficacy.

We also tested the ability of the designed miniproteins to inhibit the secondary, seeded aggregation of amyloids in vitro. Select inhibitors were first incubated with preformed amyloid fibril seeds (either AD tau, αSyn, or Aβ_1–42_). The mixtures were then added to amyloid protein monomer (tau k18+, αSyn, or Aβ_1–42_), at equimolar ratios inhibitor/monomer, and aggregation was measured by ThT fluorescence (*SI Appendix*, Fig. 8). With the addition of fibril seeds, we anticipate rapid aggregation of the monomeric protein, as the fibrils nucleate their aggregation. For both iTau-N and iαSyn-F, addition of inhibitor greatly delays this seeded aggregation, as seen by an increase in lag time in the ThT curve. For iAβ-H, the addition of the inhibitor still eliminates detectable Aβ_1–42_ aggregation.

To further validate the binding mechanism of the designed inhibitors, we introduced negative control mutations into iTau-N at the binding site of iTau-N with the tau PHF (*SI Appendix*, Fig. 9). These mutations create large steric clashes meant to disrupt binding, altering two key hydrophobic residues found within the iTau-N/PHF interface, Ala42 and Val44. Introducing mutations A42R and V44Y does not disrupt the overall computed fold of the inhibitor; however, they greatly affect the ability of iTau-N to reduce biosensor cell seeding with AD brain extract. The single A42R mutation more than halves the inhibitory capacity of iTau-N, and the double mutant A42R/V44Y completely abolishes inhibition.

Aggregated Aβ_1–42_, particularly oligomers, has been shown to be neurotoxic to neurons (49). To test whether the designed inhibitors mitigate the toxic effects of Aβ, inhibitors were incubated with Aβ_1–42_ overnight at 37 °C, then applied to cultured N2a neuronal cells, to a final Aβ_1–42_ concentration of 1 µM. A 3-(4,5-dimethylthiazol-2-yl)−2,5-diphenyltetrazolium bromide (MTT) dye reduction cell viability assay of the treated N2a cells reveals that inhibitors iAβ-H and iAβ-D can rescue Aβ_1–42_ cytotoxicity at equimolar inhibitor/Aβ ratios (Fig. 5*G*). αSyn aggregates are also toxic to neurons, and an MTT assay of N2a cells treated with 1 µM αSyn fibrils reveals that iαSyn-F can effectively rescue cytotoxicity (*SI Appendix*, Fig. 10) (9).

### In Vivo Evaluation of Inhibitors.

To evaluate the effects of the designed miniprotein in vivo, inhibitors were tested in *Caenorhabditis elegans* model strains of tau and αSyn aggregation. αSyn aggregates in *C. elegans* have been shown to have positive Congo Red staining (an amyloid-specific dye), and fast fluorescence measurements consistent with a fibrillar form (50, 51). Tau aggregates have been demonstrated to have positive thioflavin S fluorescence, another amyloid-specific dye (52). Thus, *C. elegans* serves as a useful model system for amyloid fibril inhibition. *C. elegans* strain DDP1 overexpresses αSyn fused with either YFP or CFP. Extensive amounts of aggregation can be visualized in the adult worms. Day 4 adults of synchronized cultures of DDP1 worms were administered iαSyn-F for 8 h using cationic lipids (Fig. 6*A*). The amount of aggregated fluorescent αSyn was then quantified in day 6 adults by fluorescence microscopy (Fig. 6 *B* and *C*). iαSyn-F treatment largely reduces the number of visible αSyn aggregates in the worm head regions. *C. elegans* strain BR5706 coexpresses tau with the proaggregation V337M mutation and tau F3ΔK280, an aggregation prone fragment from the tau repeat domain with K280 deleted, leading to increased levels of insoluble tau species and locomotion deficits. Synchronized cultures were treated with iTau-N at the L4 larval stage using cationic lipids for 8 h. The following day, worm locomotion was tracked and analyzed, and insoluble tau was extracted from lysed worms and analyzed by Western blot. iTau-N–treated worms showed a recovery in locomotion speed compared to vehicle-treated control (Fig. 5*D*). Anti-tau antibody staining reveals a significant reduction in aggregated tau species in the detergent soluble Radioimmunoprecipitation assay (RIPA buffer) fraction (Fig. 5 *E* and *F*). From these in vivo experiments we observe that inhibitors targeting either αSyn or tau can reduce protein aggregation and rescue subsequent locomotion deficits in *C. elegans* disease models.

## Discussion

Structure-based drug design has generated effective therapeutics for a variety of diseases, from HIV to cancer (53). However, lack of complete structural knowledge has hampered the application of this approach to amyloid diseases. In this work, we have used atomic structures of full-length amyloid proteins, determined through cryo-EM, ssNMR, and MicroED, to generate targeted inhibitor molecules capable of preventing amyloid aggregation in vitro, in cultured cells, and in vivo. Each of these amyloid structures is a product of significant advancements that have occurred recently in the field of protein structure determination. This study represents a systematic endeavor to utilize these next-generation amyloid structures for the design of protein drugs.

This work is also a product of the simultaneous advancement in the field of computational protein design occurring in recent years. As these methods continue to improve, proteins designed de novo in a computer more frequently and reliably resemble their computed folds and functions, allowing us to leverage their particular properties into biological systems (30). Here we have chosen miniproteins as the scaffold for inhibitor design because of their extremely high stabilities, amenability to high-throughput screening, and ease of modification. For example, miniproteins that bind the SARS-CoV-2 spike protein with picomolar affinity were developed within months after the start of the COVID-19 pandemic in 2020 (54).

A unique challenge with designing amyloid capping inhibitors is finding a suitable method to screen for successful designs. Traditional techniques to screen for protein binders typically involve display assays, such as yeast or phage display, to identify sequences with enriched binding. However, applying such a technique to screen for capping proteins would likely identify only sequences that tightly bind to the fibrils but not necessarily prevent their growth. Because of this, we primarily relied on computational techniques to funnel down the ∼1 million unique inhibitor sequences to a pool that could be feasibly expressed, purified, and tested experimentally. Extensive 200- to 400-ns MD simulations were useful in identifying scaffolds with good computed binding energies but were unstable for extended periods of time, as has also been described by Buchko et al. (55). Likewise, the ab initio structure prediction from primary sequences proved useful for screening out designs, which appear stable but whose sequence is more likely to adopt a completely different fold.

Additionally, our design process employed a shotgun-like approach in terms of inhibitor binding sites. Inhibitors were docked in many different positions along the fibril chain. The ranking and selection of inhibitors was done completely blind to their binding position, allowing the computed metrics to identify the optimal inhibitor/binding site pair in an unbiased manner. The binding sites for the successful inhibitors may prove useful starting points for future structure-based design approaches. Successful designs iTau-N and iαSyn-F both bind near the beta arch region on their targeted structures, suggesting this motif may be an important target. One important metric used to rank our computational designs was hydrogen-bond satisfaction. Amyloid fibrils are largely stabilized by a network of backbone–backbone hydrogen bonds running along the fibril axis, and these bonds are left unsatisfied at the fibril ends. We believe one possible reason the inhibitors are effective is their ability to satisfy these dangling H-bonds.

An important consideration with the inhibitor design approach taken in this study is the growth kinetics of amyloids. As designed, these inhibitors bind to one end of an amyloid fibril, preventing its elongation. This potentially leaves the opposite end of the fibril exposed and capable of growing. Studies of amyloid growth have demonstrated that fibrils grow primarily unidirectionally, averting this issue (56). However, conflicting reports exist demonstrating bidirectional fibril elongation as well (57, 58). Our top inhibitors do appear to be stalling amyloid aggregation and binding to the fibril ends, but future study is needed to fully understand their mechanism of action with respect to amyloid polarity. Another issue to consider is amyloid fibril polymorphism. As opposed to nearly all globular proteins, where a unique protein sequence results in a single folded structure, amyloid structures can be highly polymorphic and adopt multiple structural conformations from the same protein. An example of this is recombinant tau, which has been demonstrated to fold into several different fibril polymorphs, even within the same sample preparation. However, in a disease context, such as in AD or chronic traumatic encephalopathy (CTE), recent evidence shows that tau fibers adopt a unique fold specific to each disease. Polymorphs differ between diseases but are generally conserved within each disease process. Thus, as we gain more structural knowledge of tissue-extracted and disease-relevant amyloids, it will be important to consider this in future inhibitor design.

We demonstrate the ability of the designed miniprotein inhibitors to prevent primary amyloid aggregation, as measured through the ThT kinetics assays, as well as secondary seeding in the biosensor cell assays. There is increasing evidence that templated seeding is a primary driving force for the spread and progression of amyloid pathology in the brain, both in the case of tau and αSyn. Interestingly, comparison of the effects the tau and αSyn inhibitors have on primary aggregation versus seeding shows that the tau inhibitors that best reduce primary aggregation do not reduce seeding and vice versa (*SI Appendix*, Fig. 11). However, αSyn inhibitors that reduce αSyn primary aggregation are also effective at reducing cellular seeding. This could be a result of some underlying phenomenon that distinguishes the aggregation pathways between tau and αSyn. However, this effect may also be attributed to a difference in the protein material used, as tau ThT assays used recombinant protein while the seeding assays used patient-derived material, and the αSyn assays used recombinant material for both.

De novo designed proteins, particularly miniproteins, represent an approach for therapeutic development. Their small size, stability, and ease of expression present some benefits over established antibody therapeutics, while their adaptability and designability leverage advantages over small molecules. Further improvements to the inhibitor designs presented in this work could focus on improved binding, perhaps through protein evolution techniques, as well as methods of delivery. The eventual goal of these designed miniprotein inhibitors is to enter the brain, where amyloid fibrils are found in AD and PD. This warrants future work on delivery strategies, including fusion of successful inhibitors with cell-penetrating peptide tags or conjugation to larger delivery constructs such as brain-penetrating nanoparticles. The inhibitor designs are also genetically encodable, presenting viral delivery, another possible delivery mechanism. The treatment of neurodegenerative disease is a problem that has yet to be solved by conventional therapeutic approaches, opening a wide field for discovery and adaptation of modern techniques. Computational protein design may prove to be a useful method for addressing these challenges.

## Materials and Methods

Protein purification, preparation of AD brain tissue extract, computational design pipeline, in vitro aggregation assays, MTT cell viability assays, *C. elegans* experiments, transmission electron microscopy and nanogold binding experiments, circular dichroism and denaturation assays, cell seeding assays, and ELISA binding assays are described in detail in *SI Appendix*.

## Supplementary Material

Supplementary File

## Data Availability

All study data are included in the article and/or supporting information.
