# Supplementary material for: De novo designed protein inhibitors of amyloid aggregation and seeding
Source: Proc Natl Acad Sci U S A. 2022 Aug 15;119(34):e2206240119. doi: 10.1073/pnas.2206240119 (PMC9407671; doi:10.1073/pnas.2206240119)
Supplement: Supplementary File [file pnas.2206240119.sapp.pdf]

De novo designed protein inhibitors of amyloid aggregation and seeding

Kevin A. Murray[1], Carolyn J. Hu[1], Hope Pan[1], Sarah L. Griner[1], Jeannette T. Bowler[1], Romany Abskharon[1], Gregory M. Rosenberg[1], Xinyi Cheng[1], Paul M. Seidler[2], David S. Eisenberg[1]*

[1]Departments of Chemistry and Biochemistry and Biological Chemistry, UCLA-DOE Institute, Molecular Biology Institute, and Howard Hughes Medical Institute, UCLA, Los Angeles CA; [2]Department of Pharmacology and Pharmaceutical Sciences, University of Southern California, Los Angeles, CA.

*to whom correspondence should be addressed: David S. Eisenberg: University of California-Los Angeles 611 Charles E Young Drive, Boyer 201, Los Angeles, CA 90095; david@mbi.ucla.edu; Tel. (310) 825-3754, Fax. (310) 206-3914

Supplemental Data

Materials and Methods

Protein expression and purification

Miniprotein inhibitors were expressed using a pET28b(+) plasmid in BL21(DE3)gold *E. coli*. Cultures were grown at 37°C to an OD600 = 0.4-0.8, then induced with 0.5 mM Isopropyl β-d-1-thiogalactopyranoside (IPTG), then grown overnight at 18°C. Cells were centrifuged and pellets collected for purification. For inhibitors with solubility issues, constructs were transformed into Lemo21(DE3) *E. coli*., in which protein expression levels could be more finely tuned using optimized concentrations of L-rhamnose; transformation and induction were carried out according to the manufacturer's protocol (New England Biolabs).

Cells were lysed by sonication, and the clarified lysate ran over a Ni-NTA affinity column (lysis buffer: 50 mM Tris pH 7.5, 300 mM NaCl, 15 mM imidazole, 0.1 mg/mL lysozyme, HALT protease inhibitor cocktail, 20 mM BME). Column was washed with 10 column volumes of Wash 1 buffer (50 mM Tris pH 7, 1 M NaCl, 15 mM imidazole, 20 mM BME), followed by 20 column volumes of Wash 2 buffer (50 mM Tris pH 6, 1 M NaCl, 15 mM imidazole, 20 mM BME). Proteins were eluted and fractions collected (50 mM Tris pH 7.5, 150 mM NaCl, 500 mM imidazole, 20 mM BME). The N-terminal His-tag was then cleaved using bovine thrombin, and proteins were further purified using size-exclusion chromatography (SEC) and dialyzed into 1x PBS.

Tau k18+ (residues Q244-E380), αSyn, and $A\beta_{1-42}$ were expressed and purified as previously described[11,13,14].

Preparation of AD brain tissue extract

Brain tissue from the inferior temporal gyrus of a histopathologically confirmed case of AD (69 y.o. female; post-mortem interval time: 12.9 hours). A section of ~250 mg of tissue was cut on dry ice then homogenized in a 15 mL disposable tube using 50 mM Tris (pH 7.4), 150 mM NaCl, 1X HALT protease inhibitor cocktail. Sample was sonicated in a cuphorn water bath at 4°C, for 2 hours at 30% power. Samples were frozen at -80°C until experimental use.

Computational design pipeline

Design of the miniprotein scaffold library follows the approach previously described by Chevalier et. al[44]. Briefly, scaffolds with mixed α and β topologies (containing at least two β-strands) were designed using the blueprint format of RosettaRemodel. No disulfide bonds were allowed. Sequences for each scaffold were designed with Rosetta's FastDesign. 15,000 unique designs for each of the 7 topologies (EEEH, EEHE, EHEE, HEEE, EEH, EHE, HEE) were generated, and the top 5,000 designs from each topology were kept, filtering by overall score, β-sheet backbone dihedral angles, and number of unsaturated hydrogen bonds.

Scaffolds were docked onto the different binding sites of each amyloid structure using Rosetta MotifGraft. Scaffolds were aligned to a truncated strand of the native fibril using a backbone RMSD < 1 Å, filtering by clash score. Residues at the binding interface were then optimized, favoring the native residue sequence for each scaffold. HotSpot residues were not enabled. All Rosetta calculations were performed using the REF2015 score function with the -beta option enabled.

Stability of each scaffold after Rosetta design and optimization was assessed using molecular dynamics simulations. MD simulations were performed using GROMACS version 2018 and the CHARMM27 all-atom forcefield. Proteins were solvated in a cubic water box using periodic boundary conditions with counter ions added. Systems were energy minimized then temperature and pressure equilibrated for 100 ps. Production runs were carried out for 200 ns or 400 ns. Calculations of non-bonded interactions were gpu accelerated.

Folding of inhibitor designs were assessed with Rosetta's AbinitioRelax application, using fragments generated from the Robetta server. 50,000 trajectories were calculated for each design tested.

In vitro aggregation assays

Thioflavin T (ThT) based aggregation kinetics assays were performed in Nunc black 96 well optical bottom plates (Thermo Scientific) in a microplate reader taking fluorescence measurements every 5 minutes (FLUOstar OMEGA, BMG Labtech). All assays were carried out at 37°C in 1x PBS buffer and 50 µM ThT at a final well volume of 100 uL. For tau aggregation, 50 µM of tau k18+ (residues Q244-E380), 1 mM DTT, and 0.225 mg/mL heparin sulfate were used. For αSyn aggregation, 50 µM full-length αSyn was used. For both the tau and αSyn assays, PTFE beads (0.125-inch diameter) were used to agitate the sample, and plates were shaken at 700 rpm with double orbital rotation. For Aβ, 10 µM $A\beta_{1-42}$ was used and samples were aggregated quiescently.

3-(4,5-dimethylthiazol-2-yl)−2,5-diphenyltetrazolium bromide (MTT) dye reduction cell viability assay

Neuro2a cells (ATCC cat. # CCL-131) were cultured in MEM media (Life Technologies cat. # 11095-080) plus 10% FBS (Life Technologies cat. # 10437010) and 1% pen-strep (Life Technologies cat. # 15140122) at 37°C in 5% CO_2 incubator. Clear 96-well plates (Costar cat. # 3596) were plated with N2a cells in 90 uL culture media at 5000 cells/well and allowed to adhere to the plate for 24 hours. $A\beta_{1-42}$ samples were co-incubated in the presence or absence of inhibitor overnight at 37°C in 10 uL volume, then added to the N2a cells in triplicate (final $A\beta_{1-42}$ concentration of 1 µM). Following an incubation of 24 hours, 20 µL of Thiazolyl Blue Tetrazolium Bromide MTT dye (Sigma; 5 mg/mL stock in DPBS) was added to each well and incubated at 37°C for 3.5 hours. The assay was halted by removal from the incubator and replacement of all well media with 100 µL of 100% DMSO. Next, a SpectraMax M5 reader was used to measure absorbance at 570 nm, and a background reading taken at 700 nm which was subtracted from the 570 nm reading.

Vehicle alone treated cells were designated as 100% viable, while that treated with 100% DMSO were designated as 0% viable, and all other well readings were normalized to these values.

***C. elegans* experiments**

The following strains were acquired from the Caenorhabditis Genetics Center (CGC) and used for experiments: BR5706 (byIs193 [rab-3p::F3(delta)K280 + myo-2p::mCherry]; bkIs10 [aex-3p::hTau V337M + myo-2p::GFP]), and DDP1 (uonEx1 [unc-54::αSyn::CFP + unc-54::αSyn::YFP(Venus)]).

C. elegans strains were grown and maintained using standard conditions. Hypochlorite bleaching was used to synchronize the worms, and they were hatched overnight at 16°C in M9 media (5 g/l NaCl, 6 g/l Na_2HPO_4, 3 g/l KH_2PO_4, 1 µM $MgSO_4$), then cultured on plates with nematode growth medium (NGM; 17 g/l agar, 7.5 g/l casein, 3 g/l NaCl, 1 mM $CaCl_2$, 1 mM $MgSO_4$, 250 µM KH_2PO_4 pH 6, 5 µg/ml cholesterol) seeded with OP50 *E. coli*. Strains were maintained at 16°C. Inhibitor proteins were delivered using the cationic lipid Lipofectamine 2k. 50 uL of Lipofectamine 2k was incubated with 20 µM of inhibitor in 1x PBS to a final volume of 1 mL overnight. Worms were then added to the solution and incubated for 8 hours. Following incubation, worms were transferred onto NGM plates containing 1 µM 5-fluoro-2′deoxy-uridine (FUDR) to prevent growth of progeny.

For the αSyn strain (DDP1), inhibitors were added to day 4 adults and analysis was performed at adult day 6. Worms were mounted onto glass slides, immobilized with 100 nm polystyrene beads, and with imaged by fluorescent microscopy (GFP channel) using a ZEISS Axio Observer D1 fluorescence microscope. αSyn aggregates in the head region were counted. For the tau strain (BR5706), inhibitors were added at larval stage 4, and worms were analyzed at adult day 1. Locomotion was tracked using a Leica M205 C light microscope in 30 second intervals. Tracking data was processed using WormLab software (MBF Bioscience).

Insoluble tau levels between treated and untreated BR5706 groups were analyzed using the protocol established by Fatouros et. al. Briefly, worms were removed from plates with M9 buffer, and a 30% sucrose solution was used to remove dead animals and bacteria by flotation. Worms were then pelleted and resuspended in RAB buffer (100 mM MES, 20 mM NaF, 1 mM EGTA, 0.5 mM MgSO4). Worms were lysed in a cuphorn bath sonicator for 5 minutes at 30% power. Lysate was centrifuged at 40,000g for 30 minutes. The pellet was extracted with RAB buffer containing 1 M sucrose, then centrifuged for 20 minutes at 40,000g. The pellet was subsequently extracted with RIPA buffer (150 mM NaCl, 50 mM Tris, pH 8.0, 1% Nonidet P-40, 0.5% deoxycholate, and 0.1% SDS) and centrifuged at 40 000g for 20 min. The supernatant was then loaded onto a NuPAGE 12% Bis-Tris pre-cast protein gel and ran at 200V for 35 minutes. For Western blot analysis, iBLOT2 dry blotting system was used to transfer protein from the gel to a nitrocellulose membrane. Membrane was blocked with 5% milk in TBST for 1 hour, then washed three

times with TBST. The membrane was incubated with the primary antibody (anti-tau A0024 (Dako) 1:1000 dilution in 5% milk/TBST solution) for 3 hours, washed three times with TBST, incubated with the horseradish peroxidase-conjugated secondary antibody (goat anti-mouse IgG H and L (HRP); 1:1000 dilution in 5% milk/TBST), and washed three times in TBST. The signal was detected with Pierce ECL Plus Western Blotting Substrate (Cat # 32132), and imaging was performed with a Pharos FX Plus Molecular Imager. Actin was subsequently measured using the same protocol with a Β-Actin (C4) primary antibody (Santa Cruz Biotechnology) (1:250 dilution, 5% milk/TBST).

Transmission electron microscopy

6 uL of fibril samples (taken from *in vitro* aggregation experiments) were spotted onto Formvar Carbon film 400 mesh copper grids (Electron Microscopy Sciences) and incubated for 4 minutes. Grids were stained with 6 uL uranyl acetate solution (2% w/v in water) for 2 minutes. Excess solution blotted off, and grids were dried for 30 minutes. TEM images of fibers were taken using a JEOL 100CX TEM electron microscope at 100 kV. Nanogold experiments were imaged using a Technai T12 TEM electron microscope at 120 kV.

Nanogold particle binding

Tau k18+ fibrils seeded with AD patient extract were generated by boiling 350 µM tau k18+ in 100 mM 2-mercaptoethanol for 5 minutes in 1x PBS (pH 7.4). Sonicated AD tissue extract was added to the samples and aggregation was induced by shaking at 350 rpm at 37 ^{o}C overnight.

6 uL of the seeded fibrils were spotted onto Formvar Carbon film 400 mesh copper grids (Electron Microscopy Sciences) and incubated for 3 minutes. Grids were blotted by quickly dabbing onto filter paper, then blocked for 15 minutes with a 1% gelatin solutions in PBS. Grids were blotted and 10 uL of the primary antibody (anti-His tag, HIS.H8; 1:100 dilution in 1% gelatin-PBS) was applied and incubated for 20 minutes. Grids were washed and blotted five times. 10 uL of secondary antibody (goat anti-mouse IgG H&L (20nm gold) preadsorbed antibody in PBS) was applied for 10 minutes then blotted. Grids were then washed five times with PBS, then five time with deionized water. Grids were stained with 6 uL uranyl acetate solution (2% w/v in water) for 2 minutes, blotted, then air dried for 30 minutes. TEM images were acquired with a JEOL 100CX TEM electron microscope at 100 kV.

Circular dichroism and denaturation assay

Circular dichroism measurements were performed with a JASCO J-715 spectrophotometer in a crystal cuvette (path-length of 1mm) at a concentration of 0.20 mg/mL in 1x PBS buffer (pH 7.4). For denaturation experiments, guanidinium hydrochloride was added to protein samples with final concentrations from 0-6 M and incubated for 20 minutes before measurement. Secondary structure analysis was performed using the BeStSel server[59].

Cell seeding assays

HEK293T biosensor cells stably expressing tau-k18 or αSyn fused with YFP or CFP, developed by Diamond and colleagues at UTSW were utilized without additional authentication or characterization. Cells were grown in a humidified incubator at 37 °C, 5% CO_2 in DMEM (Life Technologies, cat. 11965092) with FBS (10% vol/vol; Life Technologies, cat. A3160401), penicillin/streptomycin (1%; Life Technologies, cat. 15140122), and Glutamax (1%; Life Technologies, cat. 35050061). Inhibitors were incubated with recombinant fibrils or patient tissue overnight in OptiMEM media before being applied to ~70% confluent biosensor cells. To seed cells, the coincubated inhibitor/fibrils were sonicated for 3 minutes in a cuphorn water bath, and mixed with a 1:20 dilution of Lipofectamine 2000 in OptiMEM for 20 minutes. 10 uL of the inhibitor/fibril + Lipofectamine mixture was added to 90 uL of cells plated in black 96-well tissue culture plates in triplicate for each concentration of inhibitor tested. Quantification of seeded aggregates was performed using a Celigo Image Cytometer (Nexcelom) in the YFP channel, imaging the entire 96-well plate. Images were processed in ImageJ, subtracting background fluorescence from unseeded cells, and using the Particle Analyzer function to count the number of particles above background. Total number of aggregates in each well was normalized by cell confluence. Standard deviation between triplicates and IC50 values for dose response curves were calculated using a nonlinear regression curve. To obtain high quality fluorescent images, a ZEISS Axio Observer D1 fluorescence microscope was used, imaging in the YFP fluorescence channel.

ELISA binding assay

100 uL of fibril samples (AD seeded tau-k19+, αSyn, $A\beta_{1\text{-}42}$; 600 ng monomer equivalent protein) was added in triplicate to Nunc black 96 well plates and allowed to adsorb overnight at 4^oC. At room temperature, sample solution was discarded by quickly inverting the entire plate, then 200 uL of SuperBlock T20 blocking buffer was added for 30 min. Blocking buffer was discarded and wells were washed with 100 uL TBST three times, then solution was discarded and replaced with various concentrations of miniprotein inhibitors in TBST for 2 hours. Plate was washed three time with TBST. 100 uL of Alexafluor 647 labelled anti-His tag antibody (1:500 dilution, in TBST) was added for 1 hour, and plate was washed three times with TBST. Fluorescence was measured using a SpectraMax M5 plate reader (ex: 650 nm; em: 671)

inhibitor	sequence	inhibitor class
iTau-A	PTRSNEYNANGDALKAAELVEKAAKELSTNADTFFFVVY	EHE
iTau-B	DKAQETADRIQTELERKNARNVTITDTVDNTFYISVHLGEFIKISVAAK	HEEE
iTau-C	PRVEQEMRGIDPQQAMTIALKLAKKLGGAMLVEVHGDTVRWTVQV	EHEE
iTau-D	DPTEDDLARKALQWAKDAPPGSTWSDTYDNRYKVTVHVGEFILVRIETE	HEEE
iTau-E	PTQHYDYRGTDIAEVARQAAELADKYNGIVTVVTRGDTIHVTVHI	EHEE
iTau-G	PRTYQTTSDNRVTVTVHIGEFILIHIDVKVPDPTATDKAAKLAEELAKT	EEEH
iTau-H	PTTYHYNLDNKVTIQVHISEFLRIRIDFDPRDMELLIRASLIAADMAAK	EEEH
iTau-K	DKAQETADRIQTELERKNARNVTITDTVDNTFYISVHLGEFIKISVAAK	HEEE
iTau-M	DPRTEEASKIATQLAKEAGPNSDVKVTYERGITIKITNGEFILIIIDIK	HEEE
iTau-N	PVHVTFHIPGVDTKQYTITDGNVAKILTELFKKMQEKANGSALVTHVIMF	EEHE
iTau-O	PTEKYKRRGDNASELHADLARKAAERGASVKLVVFGDDVEVEVHP	EHEE
iTau-P	PRRQTYNVDNNVQVHVKDNGAVHVIIITKRPDTEAIKKAAKIAETAMKT	EEEH
iTau-Q	PKTTYDRRGVDSNTATYEAAKIAAENNGTAAVYVRGDRVRIDIQ	EHEE
iTau-R	PTSTYHFKGYDVAQAAKWATDVATKRNGTTVVVLDGDTVTVHIKD	EHEE
iTau-S	PRVTIASVGGDEKKLKELAEFVAKKLNGTVETRRHGETFTFHVQG	EHEE
iTau-T	PTYHDHQRGLDAKDAVDIARKLLETYNGSVAIHIFGDTLTVRVKV	EHEE
iTau-U	APEMQKAQTTATKLAKKAQPGQTLQSNVDNKVNVTVHVGESLVIVIDAK	HEEE
iTau-V	PRTVRIERPGRFKVEVKLGESIVVYVYVESGNKEDAEEAAKLALTAIKT	EEEH
iTau-W	PRITMHFTNVDAEQIAWEAARAAAENNTAVSIFVDGNHVEVRVQP	EHEE
iTau-X	PVAKMVFYGLSEEQARKIAQKAANLSNGTVSIDSNGDTIDVTVHV	EHEE
iTau-Y	YVQVHIHFANGRTKTIKFEDGDPDEVAKKATEQAKKEANGIPVAVLVIVY	EEHE
iTau-Z	AVIIAIYIGDDETKVKKIAEDAAKALNGEYRVRTDGDTIQLTVTT	EHEE
iαSyn-A	PRKTYTSRGTQAQELATEVTKEAAKLNASIIVLVDGDTVTVHLQV	EHEE
iαSyn-B	GQHHQTYQNTDTRQVIFEAALEAAKKNADVVITQDGDTIHVDIQV	EHEE
iαSyn-C	PTERSEYKGVDATDAIRDALRIALEQNGVVVVSVHGDTVHVTFHN	EHEE
iαSyn-D	PSQRYTYKGISLDQVITIATEIAQKMGGALAIVVYGDTLSFEVHV	EHEE
iαSyn-E	PRVDIEFKNVDPFEARDVALKVAQKLGGAMAVIIDGDTIHIHIQV	EHEE
iαSyn-F	PVYHYRYKGRAAAEAAKEAAKIAQKLGGALVVRVDGDTIRITIAV	EHEE
iαSyn-G	PRSAAQYRGVDEDEVKKLAQTTAEVLNIDYKIHSDGDTITVHFQK	EHEE
iαSyn-H	DVAVTVVAGSDPREAEDWAKKVADKLGGEIRSRQDGDIVVVEVHA	EHEE
iαSyn-I	DVYVIVAKGVDEREVTKTAKKWADEANAEIRVTSDGDLVEVRVQA	EHEE
iαSyn-J	DVLVYVYNGQDPEEVEKKAKEIADKSNGKVEVDKQGDQVHVTIKV	EHEE
iαSyn-K	DVIVLVFAGYSEEDAKKIAQDTANKKGASYQTRRDGDIIVFHIHV	EHEE
iαSyn-L	DVFVYVVAGDDSTEVATKLKDEAKKQNAKFDLKRDGDIVHITIHR	EHEE
iAβ-A	PSAIYIAQGLDEDQARKIAETMSKTAGGHVEMRTDGDVIEIKLQV	EHEE
iAβ-B	PSTQETYKNTDWREVAETAEKKAREMNGTVYVEARGSTIHVTIET	EHEE
iAβ-C	PTSVVQSRGDDYDELKKKYEDWARKNNADIKVSVDGDTVRITIHM	EHEE
iAβ-D	PLEVTITIGKKIRAHAKSHQDPQLARDLKTVAEKAAKTQNLPLEVHVNNI	EEHE
iAβ-E	PVLVYKARGVDEKEARTAAKEASKALNAEVKIESDGDTFRFQVHQ	EHEE
iAβ-F	MPEAKKAIEKATEIAKKNNARIVYVEVIPGGEIHIDVKP	HEE
iAβ-G	PDITLRYRGTNAAEVIATAEDLSDKLGAIAETYSDGDTITLHLKR	EHEE
iAβ-H	PKRVTYTLNNRVHVQITHTDQKIVYVESSTGDKDAAMTAVKIADELAKK	EEEH
iAβ-I	PYTVENHKGDQSSTKITDSEDPKKAWELAMKILTEALKKNLPIFVILDQP	EEHE
iAβ-J	PTVNIAMAGSDEKTATDIAKKVADELGGELRVTTDGNAVHVHVHV	EHEE
iAβ-K	GTTFNLTTRNRIKYKFHVETDSAEDLTRVADEADKAAHNNAPFETVMVAY	EEHE
iAβ-L	PTIHETYKGYDAREVAKTAATRATKLGAEVYVELDGDTVHVRIQL	EHEE

Supplemental Table 1: Sequences and class type of designed inhibitors.

inhibitor	Amyloid protein target	Amyloid Binding site (Sequence)	Amyloid binding site (ZipperDB score)	Inhibitor binding sequence
**iTau-A**	tau	QVEVKS	-23.3	FFVVYA
**iTau-B**		GNIHHKP	-23.1	IKISVAA
**iTau-C**		VQIVYK	-25.9	AMLVEVH
**iTau-D**		VQIVYK	-25.9	ILVRIET
**iTau-E**		GNIHHKP	-23.1	IVTVVTR
**iTau-G**		VQIVYK	-25.9	ILIHID
**iTau-H**		IGSLD	-20.8	SEFLRI
**iTau-K**		GNIHHKP	-23.1	KISVAAK
**iTau-M**		VQIVYK	-25.9	FILIII
**iTau-N**		QVEVKS	-23.3	VTHVIMF
**iTau-O**		EKLDFKD	-19.5	SVKLVVS
**iTau-P**		QVEVKS	-23.3	HVIIITK
**iTau-Q**		GNIHHKP	-23.1	TAAVYVR
**iTau-R**		QVEVKS	-23.3	GTTVVVL
**iTau-S**		GNIHHKP	-23.1	PRVTIAS
**iTau-T**		EKLDFKD	-19.5	SVAIHIF
**iTau-U**		QVEVKS	-23.3	LVIVID
**iTau-V**		VQIVYK	-25.9	SIVVYV
**iTau-W**		LDNITHV	-23.8	AVSIFVD
**iTau-X**		LDNITHV	-23.8	AKMVFY
**iTau-Y**		VQIVYK	-25.9	VLVIVY
**iTau-Z**		VQIVYK	-25.9	VIIAIYI
**iαSyn-A**	αSyn	KTKEG	-22.6	ASIIVL
**iαSyn-B**		GVVHG	-24.5	ADVVIT
**iαSyn-C**		GVVHG	-24.5	GVVVVS
**iαSyn-D**		GVATVAE	-26.9	ALAIVVY
**iαSyn-E**		GVATVAE	-26.9	AMAVIID
**iαSyn-F**		GAVVTG	-26.1	GALVVR
**iαSyn-G**		AVAQKTV	-25.4	RSAAQYR
**iαSyn-H**		VVTGVTA	-23.9	VAVTVVA
**iαSyn-I**		VVTGVTA	-23.9	VYVIVAK
**iαSyn-J**		VVTGVTA	-23.9	VLVYVYN
**iαSyn-K**		VVTGVTA	-23.9	VIVLVFA
**iαSyn-L**		VVTGVTA	-23.9	VYVYVVA
**iAβ-A**	Aβ	KLVFFAE	-22.2	SAIYIAQ
**iAβ-B**		KLVFFAE	-22.2	TVYVEAR
**iAβ-C**		GVVIA	-27.8	TSVVQ
**iAβ-D**		KLVFFAE	-22.2	IRAHAKS
**iAβ-E**		KLVFFAE	-22.2	VLVYKAR
**iAβ-F**		KLVFFAE	-22.2	RIVYVEV
**iAβ-G**		VGSNK	-19.9	IAETYS
**iAβ-H**		KLVFFAE	-22.2	KIVYVESS
**iAβ-I**		GVVIA	-27.8	PIFVIL
**iAβ-J**		QKLVF	-24.0	TVNIAM
**iAβ-K**		QKLVF	-24.0	TVMVAY
**iAβ-L**		KLVFFAE	-22.2	EVYEVLD

Supplementary Table 2: Designed inhibitor targets and sequences. The amyloid protein targets for each designed inhibitor, with the sequence and ZipperDB score of each segment listed. The sequence of the inhibitor at the amyloid binding site is also listed, highlighting that the inhibitors lack any sequence similarity to the native amyloid protein.

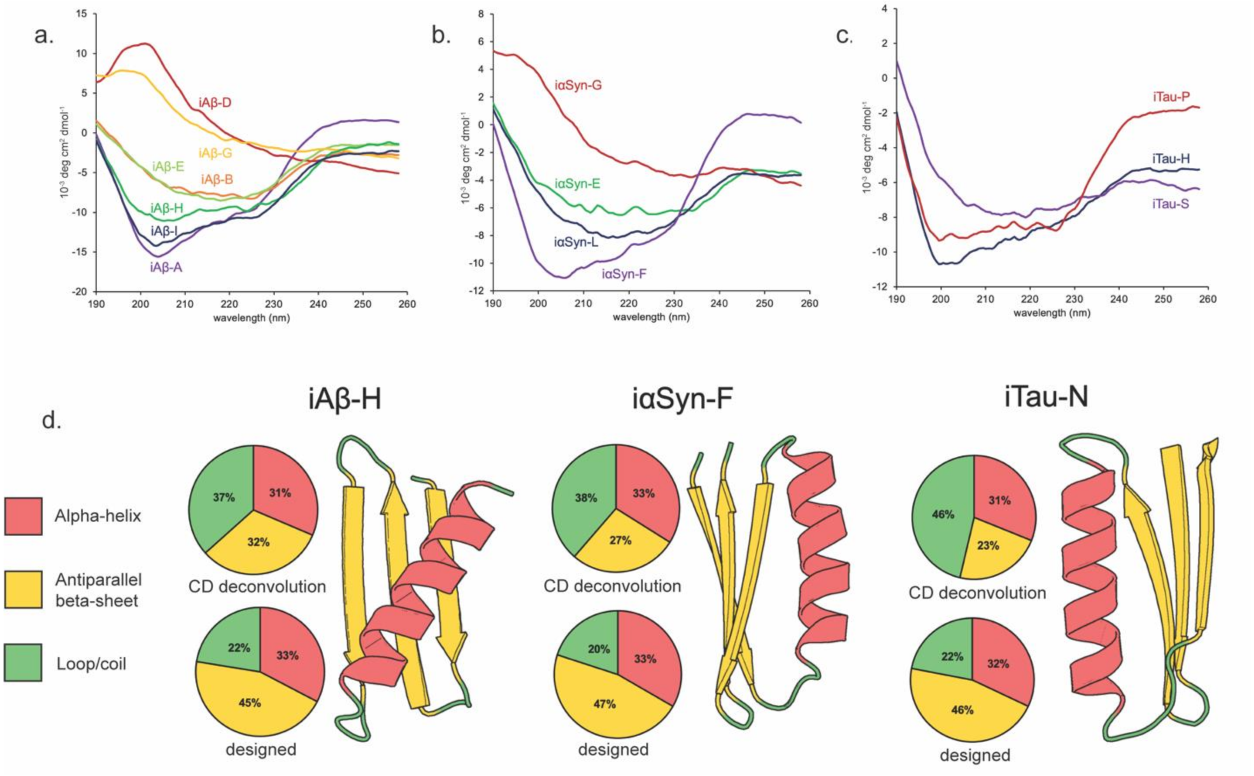

Supplemental Figure 1: CD spectra and secondary structure analysis. Circular dichroism (CD) spectra of select miniprotein inhibitors targeting amyloid-beta (**a**), alpha-synuclein (**b**), and tau (**c**). **d.** For top inhibitors iAβ-H, iαSyn-F, and iTau-N, CD spectra were analyzed to predict secondary structure composition of the purified miniproteins. Distribution of secondary structural elements for each miniprotein (alpha-helix, anti-parallel beta-sheet, and loop/coil) are show according to deconvolution of the CD spectra (top) and secondary structure composition according to the computational design (bottom). Structures of each inhibitor are shown, colored according to secondary structure. Notably, each of the inhibitors adopt a mixed alpha-helical and anti-parallel beta-sheet conformation in both the design and experimental spectra.

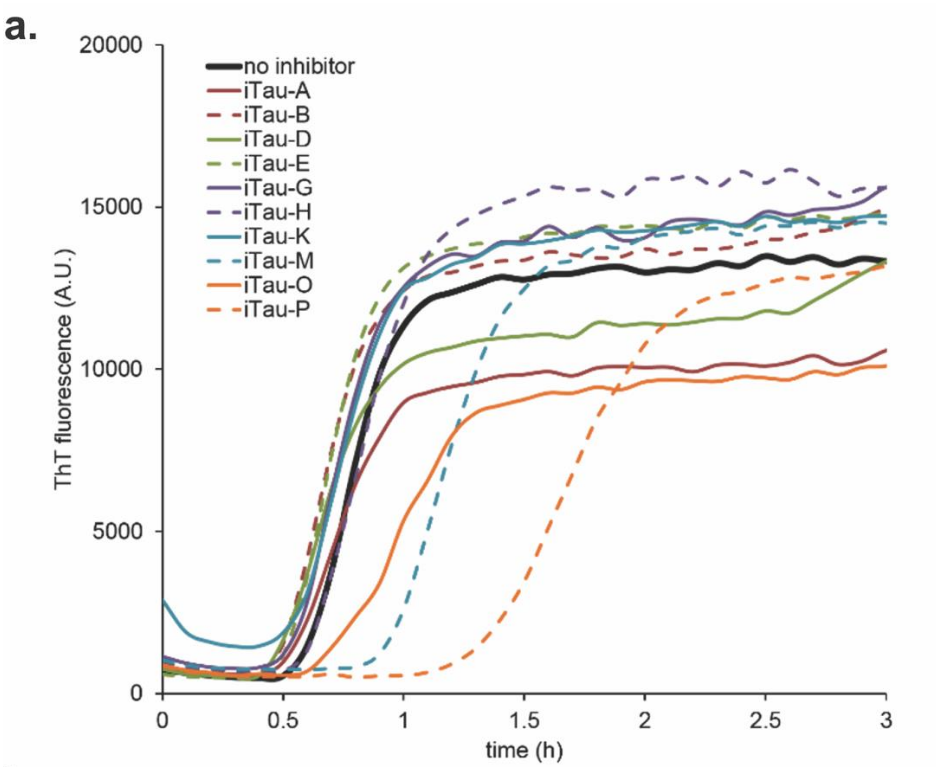

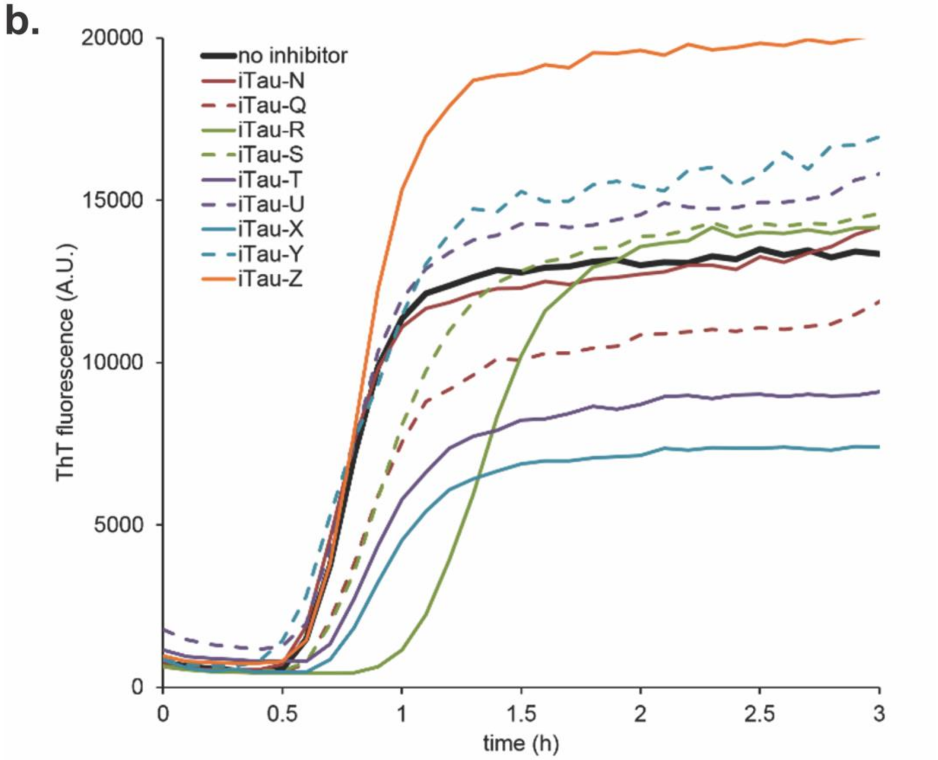

Supplemental Figure 2: Effects of designed inhibitors on primary tau aggregation. Thioflavin T kinetics assays of tau k18+ aggregation (50 μM) in the presence of 50 μM of each designed inhibitor. **a.** Kinetics curves for tau inhibitors iTau-A/B/D/E/G/H/K/M/O/P. **b.** Kinetics curves for tau inhibitors iTau-N/Q/R/S/T/U/X/Y/Z.

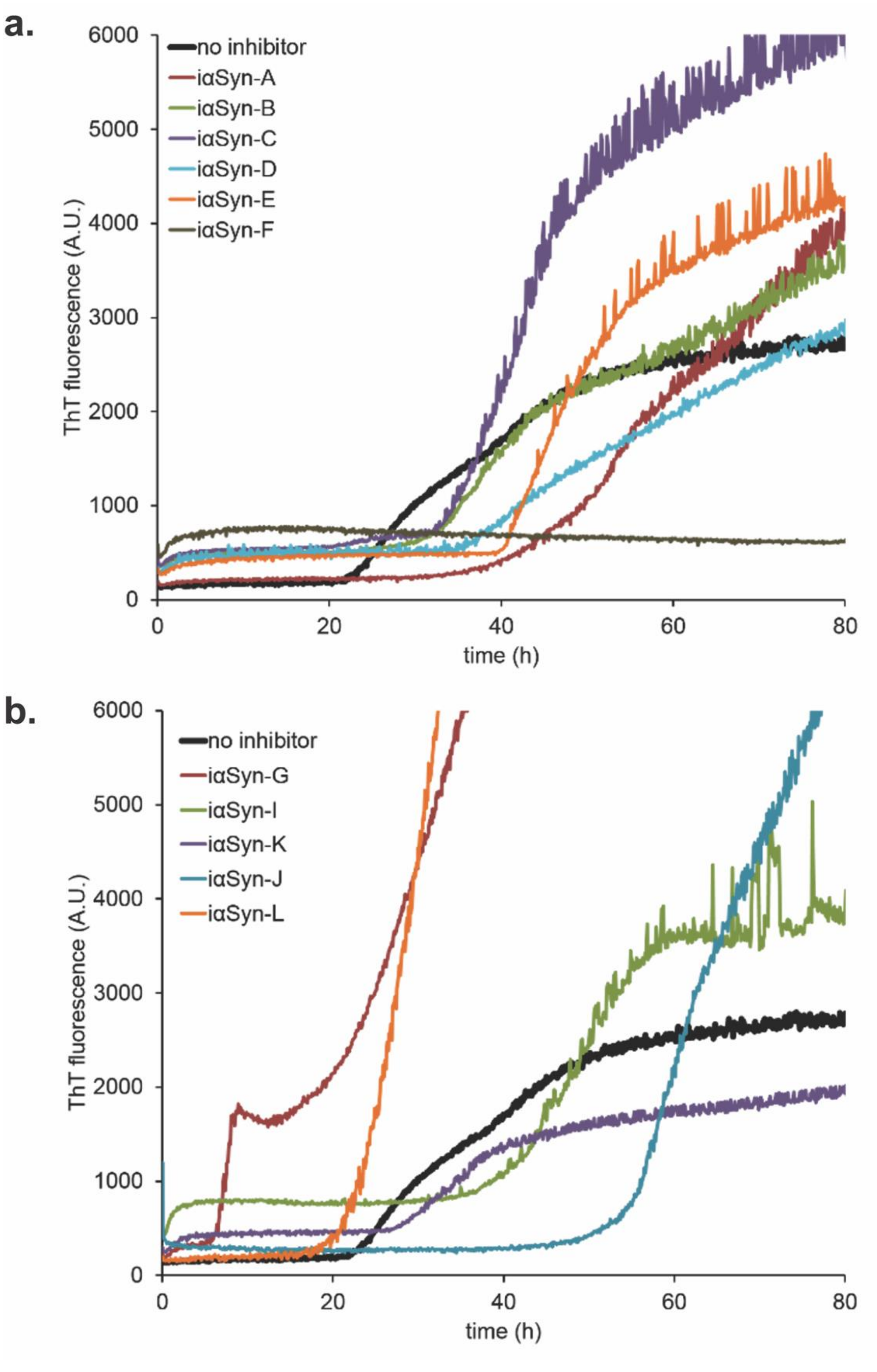

Supplemental Figure 3: Effects of designed inhibitors on primary αSyn aggregation. Thioflavin T kinetics assays of αSyn aggregation (50 μM) in the presence of 50 μM of each designed inhibitor. **a.** Kinetics curves for αSyn inhibitors iαSyn-A/B/C/D/E/F. **b.** Kinetics curves for inhibitors iαSyn-G/I/K/J/L.

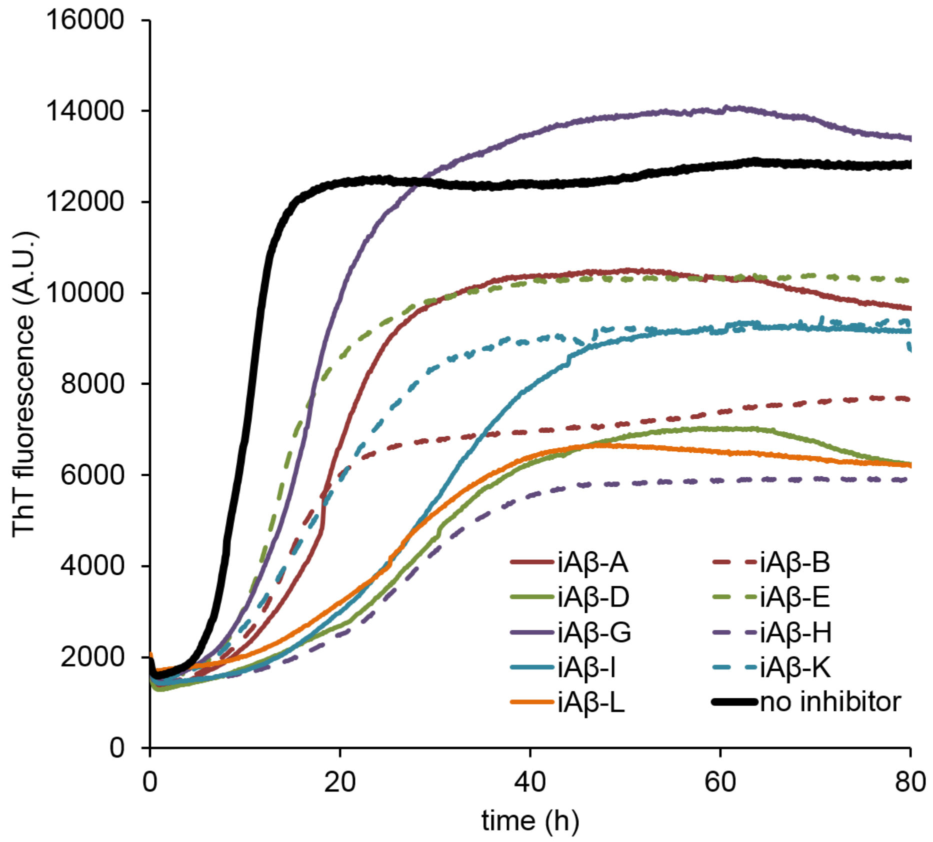

Supplemental Figure 4: Effects of designed Aβ inhibitors on primary aggregation. Thioflavin T kinetics assays of amyloid-beta aggregation (10 μM) in the presence of 5 μM of each designed inhibitor.

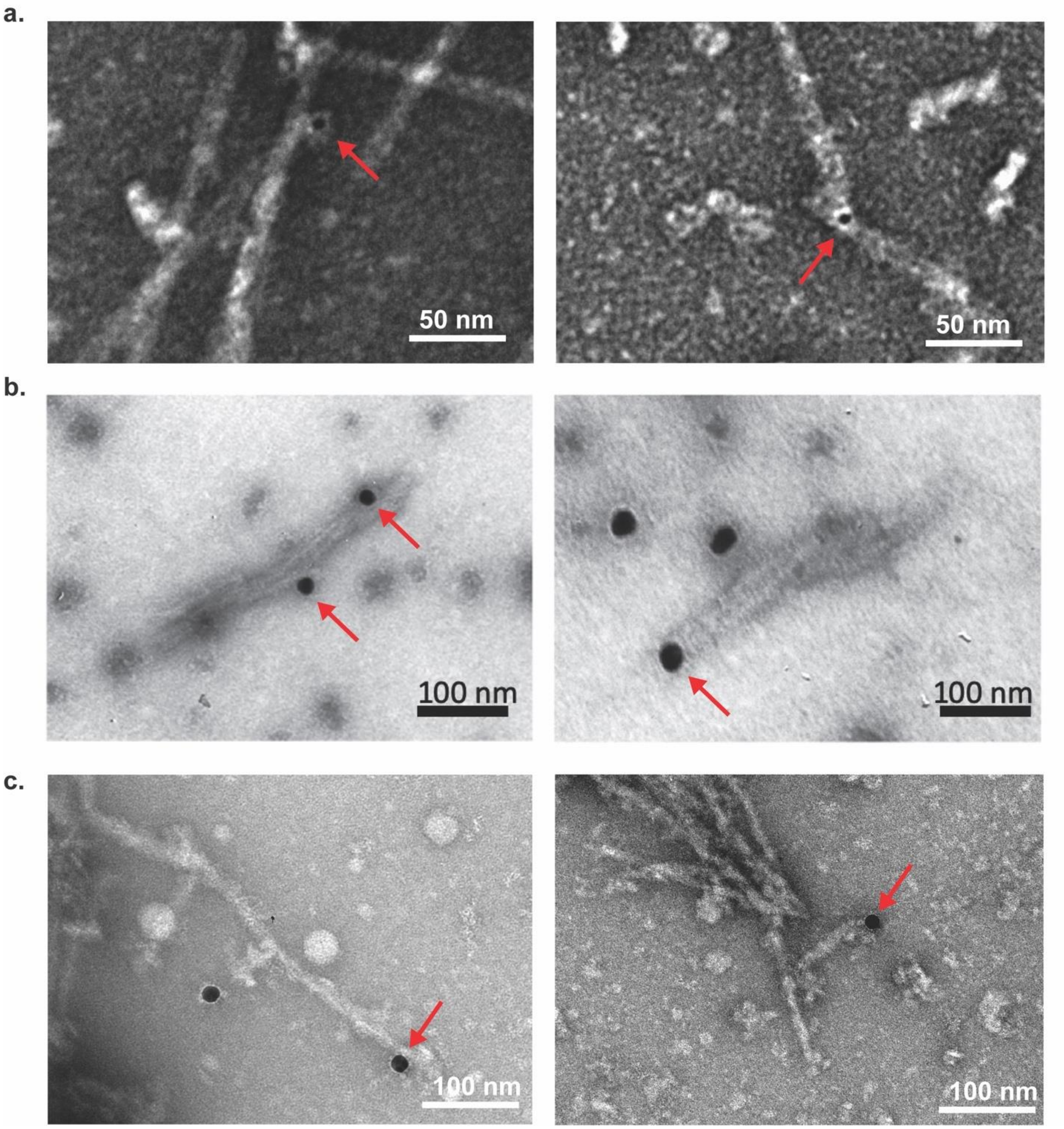

Supplemental Figure 5: Additional electron microscopy images of immunolabeled gold nanoparticles bound to inhibitors residing on amyloid fibril tips. a. iαSyn-F bound to recombinant αSyn fibrils. **b**. iTau-N bound to AD tissue seeded tau fibrils. **c**. iAβ-H bound to $Aβ_{1-42}$ fibrils. Red arrows indicate gold nanoparticles.

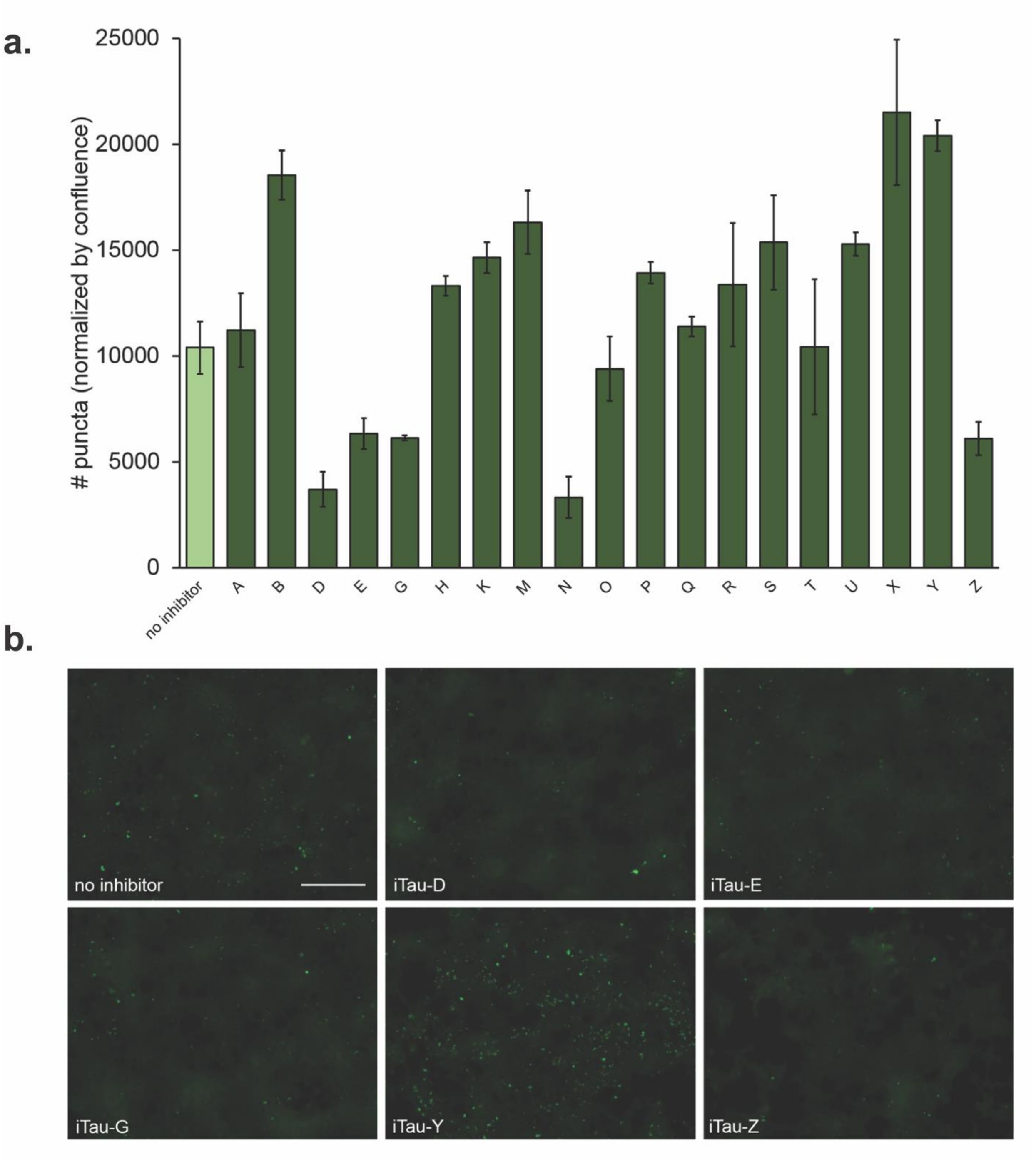

Supplemental Figure 6: Inhibition of cellular seeding by designed tau inhibitors. a. AD patient brain extract was incubated with tau inhibitors and added to HEK293T tau biosensor cells (final concentration 10 µM), and number of seeded fluorescent aggregates were quantified. Effects of the inhibitors ranged from major inhibition (iTau-N, iTau-D) to enhancement of seeding (iTau-Y). **b.** Example fluorescent images of inhibitor treated biosensor cells. Scale bar represents 10 µm.

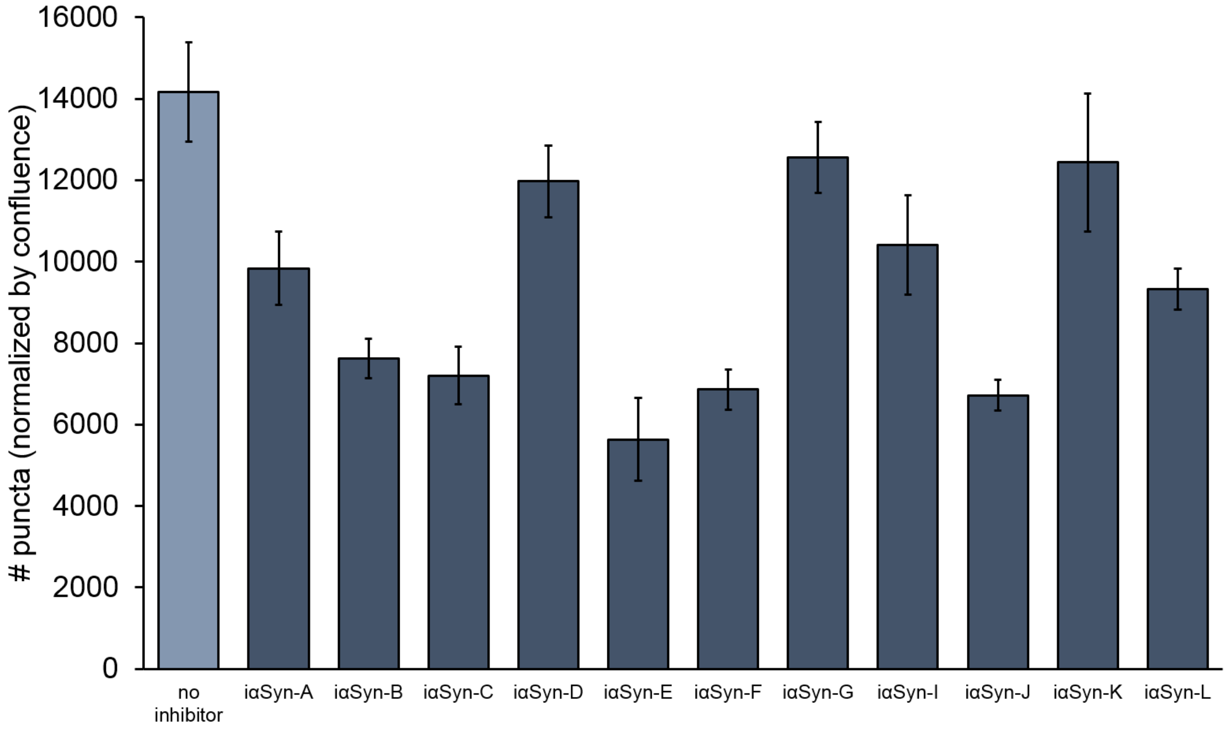

Supplemental Figure 7: Inhibition of cellular seeding by designed αSyn inhibitors. αSyn fibrils were incubated with designed αSyn inhibitors and added to HEK293T αSyn biosensor cells (final concentration 10 μM). Multiple inhibitors resulted in inhibition of seeding.

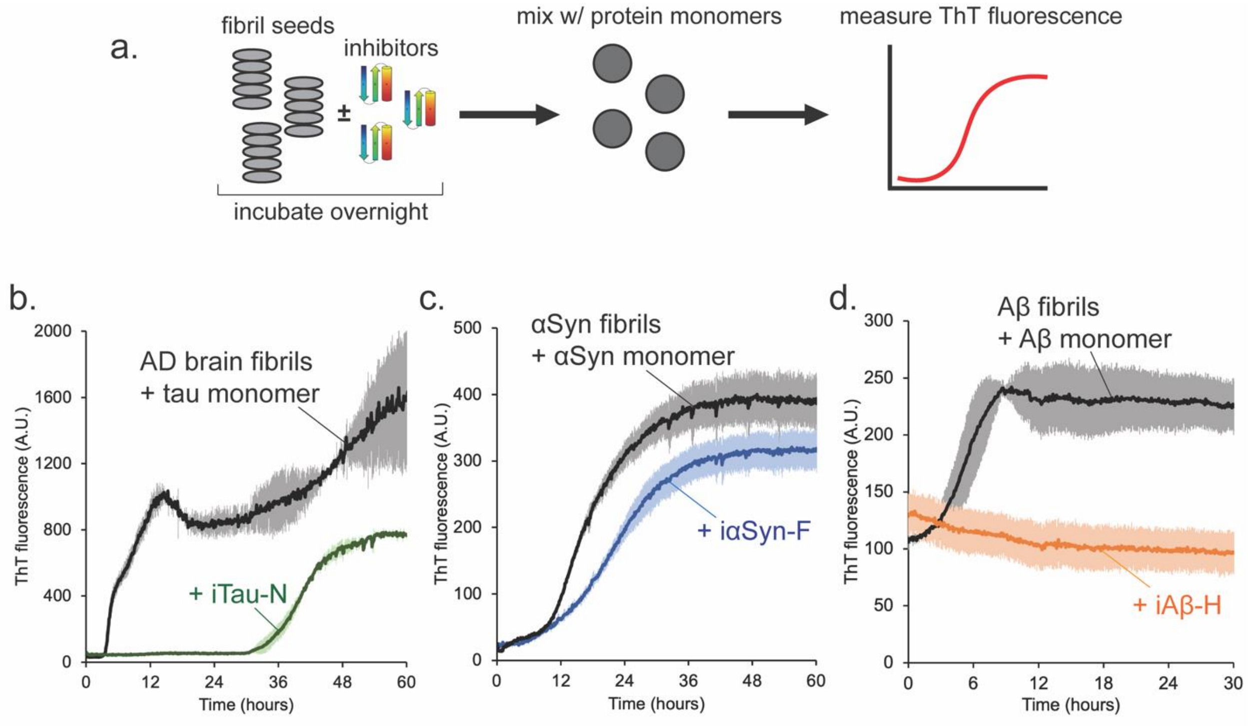

Supplemental Figure 8: Miniprotein inhibition of seeded amyloid aggregation in vitro. **a.** To assess the effects each top miniprotein inhibitor has on the secondary aggregation of amyloid fibrils, inhibitors were incubated overnight with preformed fibril seeds of the respective amyloid protein. The seed + inhibitor sample was then mixed with amyloid protein monomer, and subsequent aggregation was measured by ThT fluorescence. **b.** AD brain-derived tau fibrils were incubated with inhibitor iTau-N (50 μM), then added to tau K18+ monomer (50 μM). Without the addition of inhibitor (black curve) rapid tau aggregation is seen. However, with addition of iTau-N aggregation is greatly delayed. **c.** Similarly, iαSyn-F (50 μM) incubated with αSyn fibril seeds, then mixed with αSyn monomer (50 μM) leads to a delay in seeded aggregation. **d.** As with the non-seeded aggregation assays in Fig. 3, addition of iAβ-H completely abolishes $A\beta_{1-42}$ aggregation, even with the presence of preformed fibril seeds.

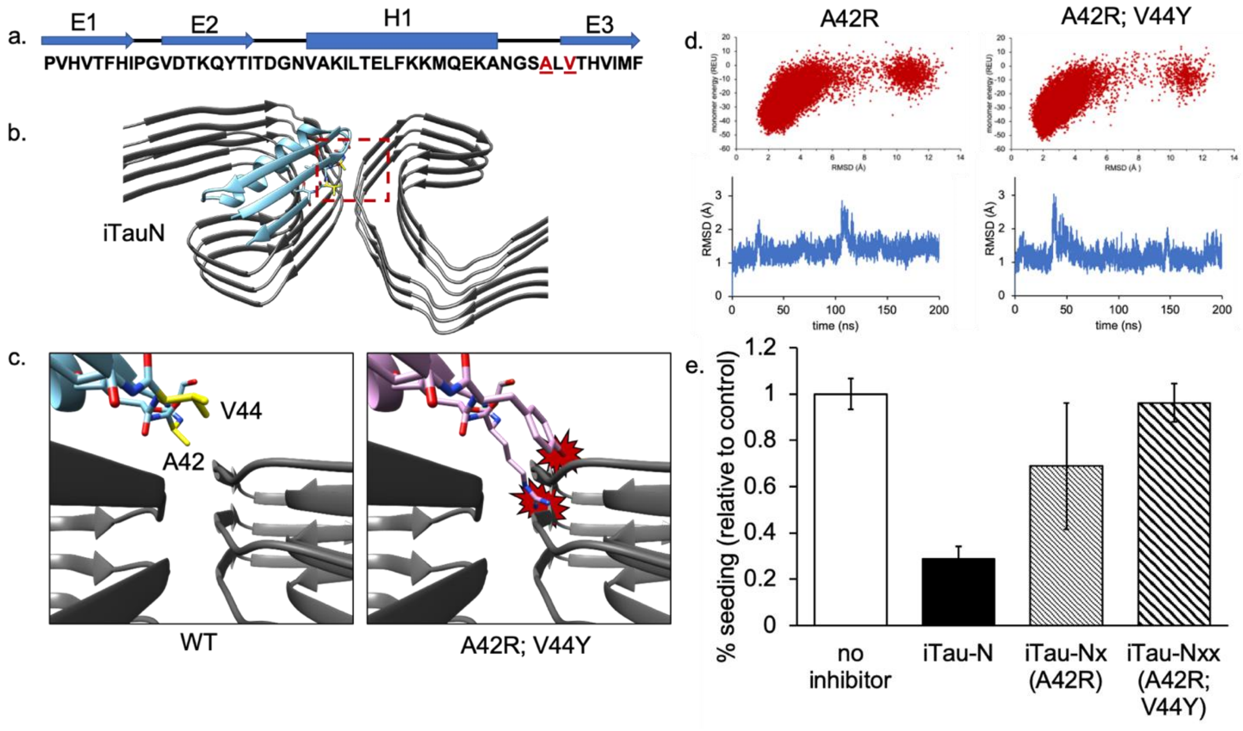

Supplemental Figure 9: Steric clashes introduced into the iTau-N/tau AD fibril binding site reduce seeding inhibition. a. The sequence of iTau-N, highlighting two key residues found in the binding interface with the tau PHF fibril, A42 and V44 (red/underlined). **b.** Model of iTau-N bound to tau PHF fibril. Red box indicating binding interface region containing residues A42 and V44. **c.** Mutations A42R and V44Y both introduce bulky steric clashes into the binding interface. **d.** Both the single and double mutant forms of iTau-N maintain stable folds, as computed by *ab initio* structure prediction calculations (top) and long-range MD simulation (bottom). **e.** Comparison of biosensor cell seeding between iTau-N and the two control mutants, iTau-Nx and iTau-Nxx. For iTau-Nx, addition of the single A42R mutation greatly reduces its ability of to inhibit tau seeding as compared to non-mutated iTau-N. For iTau-Nxx, double mutation A42R and V44Y completely abolish seeding inhibition.

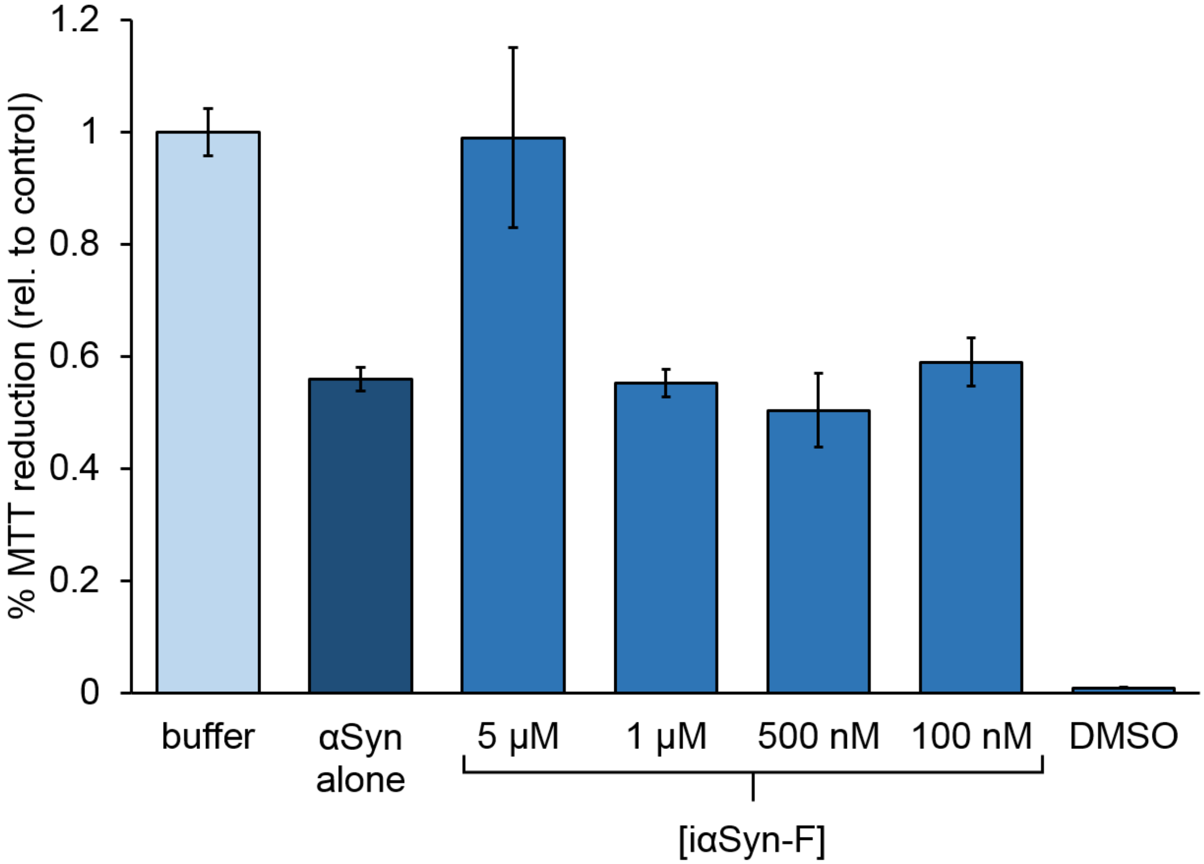

Supplemental Figure 10: iαSyn-F rescues αSyn fibril toxicity. N2a neuronal cells treated with αSyn fibrils and increasing concentrations of iαSyn-F. Cells treated with only αSyn fibrils ("αSyn alone") show ~40% decrease in cell viability as measured by MTT reduction. Incubation of fibrils overnight with 5 µM iαSyn-F completely rescues the fibril-induced cellular toxicity.

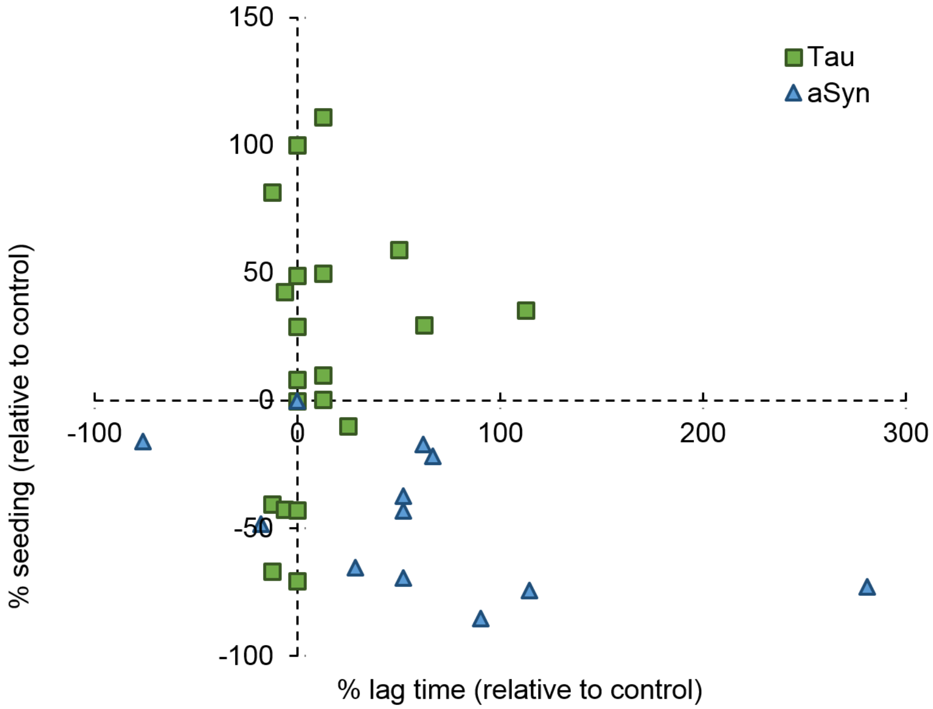

Supplemental Figure 11: Effects of designed tau and αSyn on primary aggregation and cellular seeding. Tau and αSyn inhibitors designed in this study were primarily assayed in two ways: i) their ability to reduce primary amyloid aggregation, as measured by shift in aggregation lag time in ThT kinetics; ii) their ability to prevent the seeded aggregation of protein monomer in biosensor cells. Tau inhibitors (green squares) have significant effects on either primary aggregation *in vitro*, as measured by changes in aggregation lag time (x-axis) or seeded aggregation in biosensor cells (y-axis). αSyn inhibitors (blue triangles) appear to affect both processes, with individual inhibitors reducing both primary aggregation and seeded aggregation simultaneously.
